# Categorizing the geometry of animal diel movement patterns with examples from high-resolution barn owl tracking

**DOI:** 10.1186/s40462-023-00367-4

**Published:** 2023-03-21

**Authors:** Ludovica Luisa Vissat, Shlomo Cain, Sivan Toledo, Orr Spiegel, Wayne M. Getz

**Affiliations:** 1 Department Environmental Science, Policy and Managemente, University of California, Berkeley, Berkeley, CA 94720 USA; 2 School of Zoology, Faculty of Life Sciences, Tel Aviv University, Tel Aviv, 69978 Israel; 3 Blavatnik School of Computer Science, Tel Aviv University, Tel Aviv, Israel; 4 School of Mathematics, Statistics and Computer Science, University of KwaZulu-Natal, Durban, KwaZulu-Natal 4000 South Africa

**Keywords:** Hierarchical movement path segmentation, Biotelemetry, Diel activity routines (DARs), Canonical activity modes (CAMs), cluster analysis, ATLAS tracking system

## Abstract

**Background:**

Movement is central to understanding the ecology of animals. The most robustly definable segments of an individual’s lifetime track are its diel activity routines (DARs). This robustness is due to fixed start and end points set by a 24-h clock that depends on the individual’s quotidian schedule. An analysis of day-to-day variation in the DARs of individuals, their comparisons among individuals, and the questions that can be asked, particularly in the context of lunar and annual cycles, depends on the relocation frequency and spatial accuracy of movement data. Here we present methods for categorizing the geometry of DARs for high frequency (seconds to minutes) movement data.

**Methods:**

Our method involves an initial categorization of DARs using data pooled across all individuals. We approached this categorization using a Ward clustering algorithm that employs four scalar “whole-path metrics” of trajectory geometry: 1. net displacement (distance between start and end points), 2. maximum displacement from start point, 3. maximum diameter, and 4. maximum width. We illustrate the general approach using reverse-GPS data obtained from 44 barn owls, *Tyto alba*, in north-eastern Israel. We conducted a principle components analysis (PCA) to obtain a factor, PC1, that essentially captures the scale of movement. We then used a generalized linear mixed model with PC1 as the dependent variable to assess the effects of age and sex on movement.

**Results:**

We clustered 6230 individual DARs into 7 categories representing different shapes and scale of the owls nightly routines. Five categories based on size and elongation were classified as closed (i.e. returning to the same roost), one as partially open (returning to a nearby roost) and one as fully open (leaving for another region). Our PCA revealed that the DAR scale factor, PC1, accounted for 86.5% of the existing variation. It also showed that PC2 captures the openness of the DAR and accounted for another 8.4% of the variation. We also constructed spatio-temporal distributions of DAR types for individuals and groups of individuals aggregated by age, sex, and seasonal quadrimester, as well as identify some idiosyncratic behavior of individuals within family groups in relation to location. Finally, we showed in two ways that DARs were significantly larger in young than adults and in males than females.

**Conclusion:**

Our study offers a new method for using high-frequency movement data to classify animal diel movement routines. Insights into the types and distributions of the geometric shape and size of DARs in populations may well prove to be more invaluable for predicting the space-use response of individuals and populations to climate and land-use changes than other currently used movement track methods of analysis.

## Introduction

The field of movement ecology focuses on the causes, patterns, consequences and mechanisms of organismal movement, analyzed primarily using relocation data, often collected in association with remotely sensed landscape data [1–3]. These data are then used to address questions of why and how animals move and when and where they go [4, 5]. To address these questions in the era of big data [3], a framework is required for classifying the movement tracks of animals, as well as segments of such tracks at various spatiotemporal scales [6, 7].

Although some segments of animal movement tracks may be usefully classified using a random or stochastic walk typology [8–10], ultimately many segments need to reflect both underlying landscape structures and the motivation of individuals in the context of their emergent behavioral ecology. It has been proposed that the lifetime track (LiT) of any individual can undergo a hierarchical segmentation process ([6], also see Glossary) that parses LiTs into segments of various lengths, based on two pivotal concepts: fundamental movement elements (FuMEs) and diel activity routines (DARs). FuMEs are elemental biomechanical movements and constitute a set of building blocks out of which all movement track segments are constructed (much as DNA is constructed from pairs of nucleic acids). Although the life-history of many animals are strongly impacted by seasonal and even lunar rhythms [11], DARs are the biological anchors of an ecological analysis. This follows because they have a temporal duration fixed by the diel clock that regulates the physiology and related behaviors of almost all animals, apart from species inhabiting extreme polar regions, the deepest caves or greatest ocean depths [12–14].

In this paper, we propose a method for categorizing DARs in a way that should prove useful to building models of how individuals may respond to changes in their environment (climate and landscape). This method takes cognizance of the central and anchoring role that DARs play in a hierarchical deconstruction of the LiT of an individual [6]. If these DAR segments can be related to environmental factors, then *a reassembling of DAR segments under different environmental factors* provides a way to anticipate how the structure of LiTs may respond to environmental change. In particular, these DARs can be strung together in ways that anticipate how multi-day (e.g., migrations) to seasonal-length lifetime movement phases (LiMPs) may respond to environmental change, with strings of these responding LiMPs then assembled to predict how the LiTs themselves may change.

**Fig. 1:**
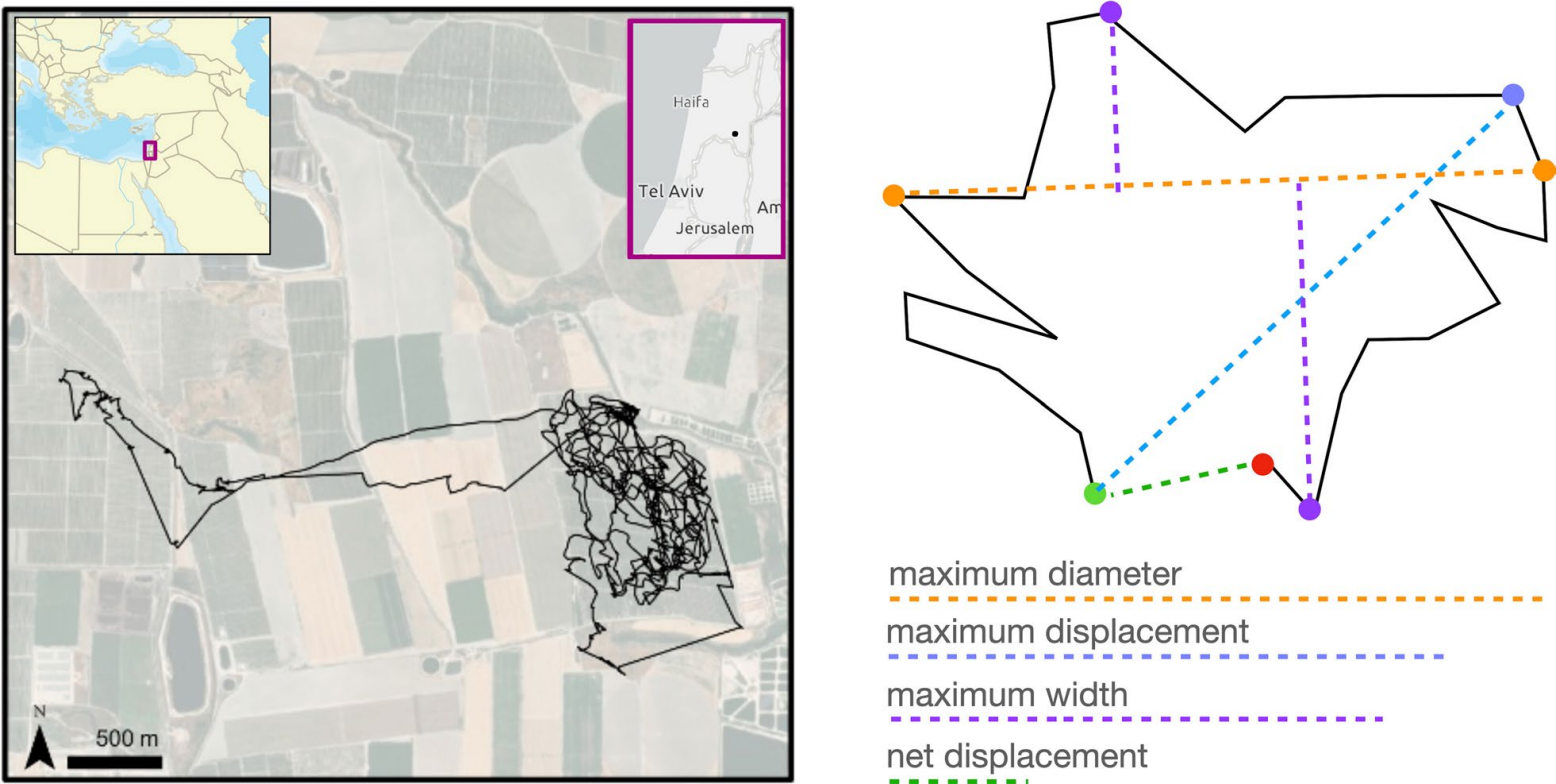
An example of a real DAR laid over the landscape where it occurred (left panel). The insets are used to depict the geographic location of our Harod valley study area. The plot uses Israeli Transverse Mercator (ITM) projection coordinates. The coordinate time series data is then used to compute the measures listed below, as illustrated using an imaginary trajectory (right panel). The whole-path metrics we used were: the maximum displacement from the green starting point (blue dashed segment), the maximum diameter (maximum distance between any two points; orange dashed segment); the maximum width (sum of the two purple dashed segments, which uses the points furthest on either side of the orange maximum diameter line), and net displacement (the green dotted line which is distance between the green start and red end points), which provides a sense of “DAR openness”

At each hierarchical level, we need to categorize the different types of segments that may occur—their length, amount of space covered, and various modes of movement. At the canonical activity mode (CAM) level (e.g., corresponding to behavioral modes of searching, resting, or heading towards desired locations) [15–18], several or many of which constitute one DAR [6], various local path metrics (i.e., computed using a sliding window along the relocation data time series) are likely useful for discriminating among CAM types. Examples of such metrics include tortuosity, distance moved, and area searched. At the DAR level itself geometric whole-path metrics that are relatively insensitive to data resolution (relocation frequency; [19–21]), such as various types of width, breadth and displacement measures, may be more useful than those used to characterize CAMs. Here we propose and demonstrate the application of four geometric whole-path metrics (Fig. 1) that can be used to characterize the relative size, the degree of elongation, and the openness (with respect to start and end points being close or far from one another) of different DARs. These metrics can also be used, if desired, to assess the orientation of the trajectory with respect to its end points (viz., in relatively closed DARs the start/end locations may lie either to one side of the trajectory or may be more centrally located).

We note, because we are interested in characterizing a daily track using a single DAR rather than with a sequence of several or many CAMs, that the measures we use are all scalar (1-dimensional) distance metric characterizations of the geometry of the track (see Fig. 1). For applications where the space filled by the track is important, 2-dimensional area-related metrics are more appropriate: for example, in classifying multi-day LiMPs or even complete LiTs, where we may be contrasting central place foraging, territorial, migratory and nomadic movement syndromes [22, 23].

As with many analytical methods in movement ecology [24, 25], the method we propose here for categorizing DARs will exhibit some sensitivity to the relocation frequency of the data used. Thus, we recommend using data that has a sub-hourly or multi-minute frequency (e.g., 2–20 points an hour). In organizing the data for DAR analysis, we need to decide where to break multi-day tracks into 24-h diel segments: the most appropriate start/finish points will depend on the daily rhythms of the movement of individuals within a population (e.g., in the context of rhinos see [26]). Once this is done, our method then employs a hierarchical clustering algorithm to identify a set of *n* cluster types [27, 28]. The number of clusters is determined using various heuristics [27] that recognize that the optimal choice is actually a subjective trade-off among several criteria. These include: capturing a desired level of the variation when the DARs are organized into descriptive spaces of lower dimension than the factor space itself; having sufficiently many categories to reveal novel phenomena that may be masked if particular categories are not separated out; and, having few enough categories so that the number of DARs are sufficient to generate reliable average statistics in all categories. We stress, however, that any reliable cluster analysis suffices for our method, including various machine learning methods [29, 30].

In addition to using hierarchical clustering to categorize our DARs, we also conducted a PCA [31]. This analysis is typically used to reduce the dimensionality of data, while minimizing information loss, by generating a set of uncorrelated factors [32], the factors themselves often having meaningful interpretations (e.g., see [33]). A PCA is most useful when the number of variables underpinning the data is large (e.g., a dozen or more). When this is not the case, as in our study, a PCA may still be useful because the first PCA factor (PC1) represents the “best composite of comparable measures” (in the sense of explaining more variation than any other composite of these measures) for implementing regressions and other types of statistical analyses. We demonstrate this here, using a generalized linear mixed model (GLMM) approach to evaluate the effect of age and sex on DAR size. In addition, when the first two PCA factors (PC1 and PC2) together account for most of the variance, then we can plot the data in a two dimensional space with these factors as axes without much lost of information.

In applying our methods to real data, we might expect that the diel activity routines (DARs) of individuals varies seasonally [34, 35] or even with phases of the moon [36, 37]. We should also expect some day-to-day variation in the DARs of many animals due to daily vagaries in the acquisition of resources needed to sustain life. Additionally, we should expect these patterns of variation to differ among individuals reflecting to some extent different personality types (e.g., risk prone versus risk averse; [22]), although we expect environmental factors to be important drivers of DAR variation [38].

We develop and present our method using movement data obtained by sampling the flight paths of Barn owls (*Tyto alba*) ([39, 40]), tracked by an ATLAS (Advanced Tracking and Localization of Animals in real-life Systems) installed in the Harod valley, Israel. Since barn owls have an eclectic diet (they are nocturnal predators of small vertebrates, primarily rodents inhabiting both sylvan and agricultural habitats [41, 42]), we anticipated identifying several different types of DARs in these data, representing a diversity of movements, spatial locations, and size of foraging bouts.

Our method allowed us to identify 7 DAR categories that we then used to assess an individual’s temporal implementation of different DARs and to compare DAR distributions across groups of individual based on various factors (sex, age, location). We anticipated, for example, that we might be able to distinguish among explorers (larger, possibly open DARs) and exploiters (smaller likely closed DARs) across similar environments, as well as among individuals within families [43]. Finally, we note with hindsight that though most of the DARs we identified in our owls were lopsided with respect to DAR start/end points (leading to a very high correlation between two of our measures), which is not unexpected for central place foragers, all four measures are needed if our method is to be generally applied to sets of DARs that include tracks moving to both sides of identified start/end points (as in the right panel of Fig. 1).

## Methods

### Study site and species

We tracked barn owls (*Tyto alba*) using an ATLAS (Advanced Tracking and Localization of Animals in real-life Systems) installation in Israel’s Harod valley (32$^\circ$ 30’ N 35$^\circ$ 29’ E; Fig. 1, left panel). This valley is a mosaic of intensive agricultural landscapes (field crops, fishponds, and orchards), rural settlements, and open natural areas. Barn owls are cavity nesters considered as an effective pest-control agent mitigating rodent outbreaks in agricultural fields. Thus, dozens of nesting boxes have been deployed through the region over the last three decades to mitigate nest site limitations and enhance the local resident populations [44]. Owls have been captured in these nest boxes during the day (inactivity period), as part of the ongoing monitoring. Individuals were banded with metal rings for individual identification, measured (wing length, body mass), aged, and sexed (feathers were taken for sex testing in the lab). Mature fledglings and adults in good body condition were fitted with tracking devices attached with a Teflon harness in a backpack or leg-loop configuration. Total mass of an ATLAS tag and harness is $13 \pm 1$ g, which is $<4.3\%$ of the body mass of the smallest tagged individual. Trapping and tagging procedures were authorized by permits 2019/42155 and 2020/42502 from Israel Nature and Parks Authority.

### Data collection and processing

The ATLAS system is designed to collect movement data at the resolution of seconds and higher. This system includes radio transmitters with unique tag-IDs, a set of ground stations with tower-mounted antennas, and central data-processing and storage servers [45, 46]. The ground stations receive tags’ transmissions at ca. 1/4 or 1/8 Hz (depending on settings) and if received by $\ge$3 ground stations locations are computed at high accuracy (±5m) from differences in the 3 or more signals’ time of arrival [40, 47]. The ATLAS reverse-GPS approach (localizations are estimated at the receivers’ side, while in GPS by the tags themselves) allows tags to be inexpensive and lightweight compared with standard GPS approaches, thereby providing many more data-points per unit weight of tag compared to other wildlife tracking systems [3]. These features make this system particularly effective in collecting high resolution and accurate data over extended periods (months) and therefore appropriate for investigating diel activity routines (DARs) simultaneously across many individuals.

During 2020 and 2021 we tagged 92 owls. To ensure data quality we filtered out localizations and smoothed trajectories according to a previously described pipeline [48]. Specifically, we excluded fixes with low system-accuracy estimate (STD > 50), or those requiring movement speed > 15 m s$^{-1}$ from a preceding location. Collected data points were assigned an (X,Y) value using Israeli Transverse Mercator (ITM) coordinates and dateTime stamps. Due to the nocturnality of the owls, we segmented the smoothed trajectories to nights (i.e., giving individual night.IDs) by segments from 10 a.m. to 10 a.m. the following day. The function AdpFixedPoint from the R package toolsForAtlas was used to assign string of locations in each such DAR into either *move/flying* or *stop* segments. Move/flying segments were associated with a seg.ID value.

We then identified all individuals in our data set with > 30 complete nights (i.e., comprising > 1000 successful localizations per night) to obtain a sample of 44 birds tracked over $142 \pm 103$ (mean ± sd) nights. From these data we extracted DARs by grouping data by night ID and selecting the ones with data collection (of a complete night) started before 9 p.m. and ended after 2 a.m. of the following day. Although we rejected DARs with $<\,1000$ points, it is still possible that some of our DARs had missing points because the individual moved outside of the range of the ATLAS sensing area. In such cases, the possibility exists for the DAR to be misclassified as belonging to a smaller excursion type than it really should. We did not undertake a thorough search for such cases because of our methodological rather than ecological focus.

For each of the 6230 DARs so obtained, we extracted first and last flight segment using seg.ID and then calculated the initial point and the end point of the DAR. The latter were obtained by calculating the average coordinates considering the *n* points ($n=20$ in our case) before the first flight segment to extract the initial point and the *n* points after the last flight segment to extract the end point. Also, the data we collected were at a higher relocation frequency (i.e., 4 or 8 s intervals) than needed for our analyses. Thus, we subsampled our data down to a frequency of one point every 5 min and compared these with 2 min, as well as 10 min, subsamples to ensure that our results were insensitive to this subsampling choice (e.g., see Additional file 1: Fig. B.1 where sensitivity of our measures to this subsampling is discussed in the captions of Additional file 1: Figs. B.2 and B.3 in Appendix B). For each of the DARs so obtained, we computed the following four measures (Fig. 1) for the purpose of DAR categorization: net displacement, bee-line distance between start and end points of each DAR; maximum displacement, distance from the starting point to the most distant point in the DAR data; maximum diameter, the greatest distance between any two points on the trajectory, with the line segment joining these two points referred to as the diameter; maximum width, the sum of the maximum distances of the trajectory points from either side of the diameter line. We chose these metrics because they are much less sensitive to variation in sampling rates than distance-traveled-along-the-track or turning angles [6], and they capture the spatial geometry of the DAR as a whole, including extent (or scale), elongation, and openess.

### Clustering and factors

We used the R packages stats (in particular functions dist, hclust and cutree) and factoextra (function fviz_nbclust) to perform the hierarchical clustering of DARs characterized by the four measurements listed above. This was done after removing any rows presenting NAs in the resulting dataframe of measurements. These NAs were mostly connected to the net displacement calculation, because flight segments could be detected right at the start or at the end of the DAR, thereby not providing enough points to calculate the average coordinates for the start/end point. We normalised values by computing the z-score transformation of the variables using the R function scale. We proceeded with a cluster analysis, using the R package stats.

**Fig. 2:**
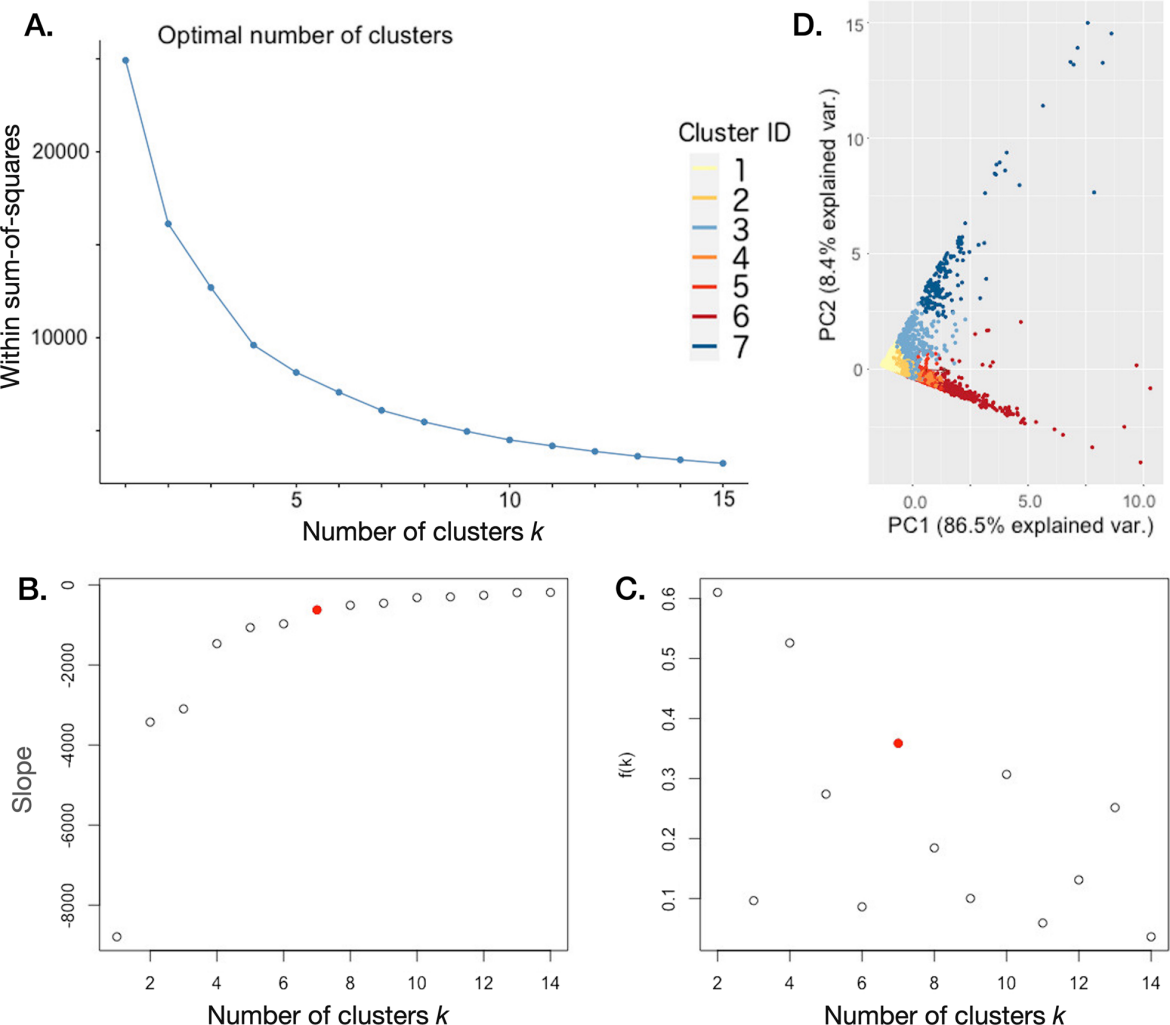
**A** A plot of the within sum-of-squares as a function of the number of clusters *k* (denoted *wss*(*k*)). The so-called elbow method can be used to determine the number of clusters, if a point on this graph exists that looks like an elbow (i.e. dramatic change in slope). **B** A plot of the slope of the line *s*(*k*) between the points *wss*(*k*) and $wss(k+1)$. **C** A plot of the relative change *f*(*k*) in the slope *s*(*k*) (i.e., $f(k) = \frac{s(k-1)- s(k)}{s(k-1)}$). *Note*: the relative change of slope is highest for $k=2$, then for $k=4$ and next for $k=7$ (point shown in red). **D** A plot of the results of our cluster analysis in the 2-dimensional space spanned by the two principal components of a PCA, which together explain 94.9% of the observed variation

The number of DAR clusters $k$ that we use to compare DAR distributions among individuals grouped by type (i.e., age, sex, or location) depends on the extent to which we trade deeper ecological insights for greater statistical power. Higher numbers of clusters provide greater potential for differentiating increasingly homogeneous syndromic movement groups [22, 53] but weaken the statistical power of the analysis through an increased number of categories that have diminished sample sizes. Various hierarchical clustering methods use the within-sum-of-squares (wss) values (a measure of the variability of the samples/observations assigned to each cluster) to select the number of categories by computing wss$(k)$ (i.e., wss as a function of the number of clusters $k$) and identifying the point at which an increase in $k$ no longer leads to a relatively strong decrease in the computed wss value. The “elbow method” is based on identifying the point $k$ on a plot of wss($k$) as function of $k$ (e.g., for our data see Fig. 2A) at which the slope $s(k)={\rm wss} (k+1)-{\rm wss}(k)$ (e.g., Fig. 2B) changes in a way that looks like an elbow [28]. If no obvious elbow can be visually identified, then the relative change in slope $f(k)=(s(k-1)-s(k))/s(k-1)$ can be plotted to identify the values of $k$ for which this change is markedly larger than its preceding neighbor (e.g., Fig. 2C).

Our computation of the change in slope $f(k)$ for our data produced several points where $f(k)$ was noticeably higher than its preceding point, namely $k = 2$, 4, 7, 10 and 13 (Fig. 2C). The value $k = 2$ provides minimal opportunities for behavioral/ecological insights, while $k = 10$ and $13$ meant that some of the clusters contained only a few observations. Thus, $k = 4$ and 7 are more obvious choices in our case for balancing ecological insights with statistical power. In a focused behavioral/movement ecology study, both options could have analyzed. Here, we opted to illustrate our methods using the more diverse case $k = 7$ but could equally have analyzed the case $k = 4$ as well if we needed to obtain stronger statistical results in comparing cluster distributions among different groups of individuals. Finally, we note that whatever value for $k$ is chosen, all conclusions that are reached are regarded as ecological differences among groups conditioned on the diversity and ecological interpretation of the types that constitute the identified clusters.

None of our measures on its own provides a reliable measure of the size of a DAR because long thin DARs (those with large max diameter but relatively small max width) will facilitate less sensory coverage of a landscape than shorter fatter DARs of the same length if the width is comparable to the visual detection range. The dominant eigenvector (PC1) of a PCA, however, is known to explain more variance than any other eigenvector of the covariance matrix of the data used in this analysis. Since the weightings of all the variables defining PC1 are positive and use the same type of measure (all length in our case; also see [33] where units are concentration), PC1 can be regarded as a composite variable in this measure that accounts for more of the variation in the data than any other composite of underlying variables. Thus we performed PCA to obtain this variation-maximization factor (PC1), using the R package stats, and then used the R package ggbiplot to visualize the results.

We used our cluster and PCA results to provide us with a sense of how the diel movement activity of individuals varied with respect to sex and age. First, we analyzed cluster distributions across individuals, time, and space. Specifically, we used demographic and temporal information to compare the cluster frequencies with respect to sex, age, and season. Barn owls, like many birds in Mediterranean climates, have a biological annual cycle that can be conveniently split into 3 quadrimesters. In the case of barn owls, the first quadrimester (February–May) corresponds to the local breeding season. The second quadrimester (June–September) corresponds to the rearing/post breeding period. The last quadrimester (October–January) corresponds to the the fall-winter season, during which owls recover and prepare for the next breeding season (see [49] for more details).

Second, we evaluated the spatial distribution of clusters with respect to starting points and also sequences of cluster types over time for each of the individuals. Third, we used the lme4 and effects R packages to implement a GLMM evaluation of the extent to which DAR size is dependent on the fixed binary variables sex (male/female) and age (young/adult). The model included two random variables: RingID to account for repeated measures from the same individuals and date to account for some expected dependence on time. We carried out this analysis with respect to a square-root transformation of the dependent composite-size variable PC1 and then, for purposes of comparison, we repeated the analysis with respect to the square-root of the partial-size variable maximum displacement. We plotted model residuals—their distributions and QQplots—to ensure a reasonable fit for normality and a lack of heteroscedasticity (see Additional file 1: Fig. C.1 in Appendix C).

## Results

### DAR clusters

**Fig. 3:**
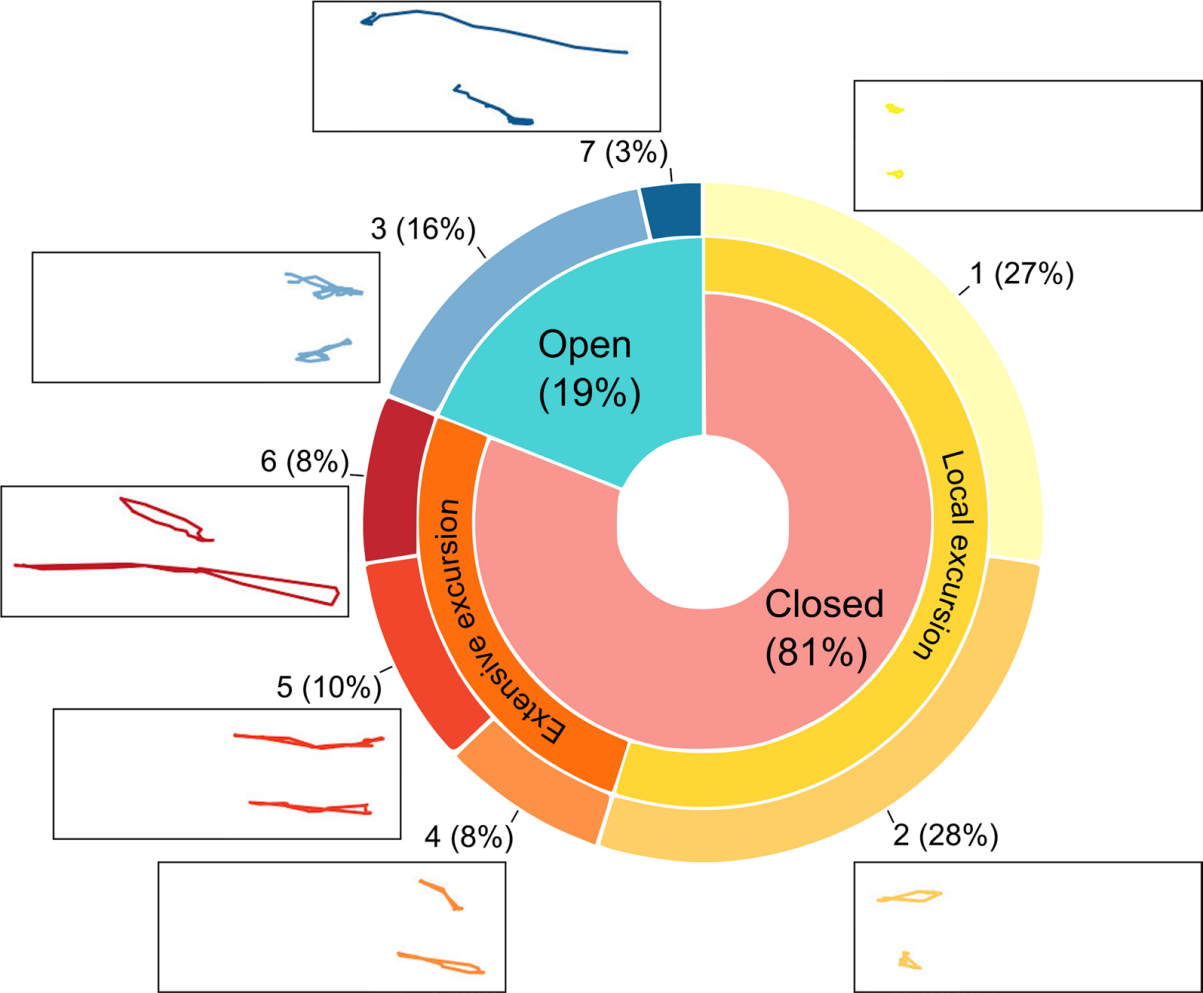
The seven panels depict two randomly chosen examples for each DAR plotted at the same scale and same north/south (vertical) and east/west (horizontal) orientations (using a 5-min subsampling to generate the plots), while the wheel depicts the proportions of each. The colors and percentages, as listed in Tables 1 and 2

As mentioned, we clustered our DARs according to their measures of net displacement, maximum displacement (from starting point), maximum diameter, and maximum width. Information on cluster sizes is provided in Table 1. From our PCA [31], we identified the first three principal components. The first, PC1, with the weights of maximum displacement, maximum diameter, and maximum width providing equal contributions and being twice as important as net displacement (i.e., a composite measure of size or “extent”) explained 86.5% of the variation. The second, PC2, with net displacement being overwhelmingly responsible for the separation of points along this axis (i.e., a measure of “openness”) explained 8.4% of the variation. The third, PC3, contrasted [maximum displacement + maximum diameter] with maximum width (i.e., a measure of “elongation”) explained 4.3% of the variation. Thus, these three principal components together explained $>\,99\%$ of the variation. We note that weak correlations ($<1/3$) exist between net displacement and the other three factors, while maximum displacement and maximum diameter are highly correlated (see Additional file 1: Eq. A.1 in Appendix A for details). This occurs when most of the DARs are highly elongated round trip flights to a particular area (e.g. types 4–6 in Fig. 3), unlike the constructed example in Fig. 1 where the maximum displacement is noticeably shorter then the maximum diameter.

**Table 1:** The results of our cluster category identification using the Ward method [50]

Cluster ID	DARs		
(color)	$$n \ (\%)$$		
1 (yellow)	1680 (27%)		
2 (dark yellow)	1719 (28%)		
3 (light blue)	972 (16%)		
4 (orange)	514 (8%)		
5 (light red)	627 (10%)		
6 (dark red)	531 (8%)		
7 (blue)	187 (3%)		

Components	PC1	PC2	PC3
Variation explained	86.5%	8.4%	4.3%
Factors			
net displacement	0.28	0.95	− 0.10
max. displacement	0.57	− 0.13	0.44
max. diameter	0.58	− 0.13	0.33
max. width	0.50	− 0.24	− 0.83
Interpretation	Extent	Openness	Elongation

In the table on the left, we list the identification numbers, cluster sizes and associated colors (to help identify clusters in figures) of the 7 identified DAR clusters. We use a yellow to red color scheme for closed DARs, and blue for partially open and wide open DARs, as described in Table 2 and depicted in Fig. 3. In the table on the right we list the four variable weightings that define the directions of our first three principal components of the PCA analysis

The 7 different types of DAR clusters we identified can be interpreted in terms of the average value of the four measures with each DAR type (Table 2). Specifically, we paid attention to the openness, size, and eccentricity of the DARs, defining transverse DARs to be those in which the average maximum diameter is considerably less than the maximum width (less than 1/4 in our case—see Table 2). Thus, among the 5 closed (i.e., returns to staring point) DAR categories we identified five excursion types: local (1, yellow), small (2, dark yellow), transverse (4, orange), medium (5, light red) and large (6, dark red). We also had one partially-open DAR category with primarily small to medium excursions with a net displacement of around half a kilometer (3, light blue; see Table 2) and one wide-open DAR category of relatively large excursions essentially representing a one way excursion, even if circuitous (7, blue; see Table 2 and Fig. 3).

In Table 1 we see that over 81% of the DARs were closed, 16% partially open, and only 3% were wide open (i.e., essentially one-way excursions). Around 55% of the closed DARs were local (average diameter $~\,0.8$ km) or relatively local excursions (average diameter $~\,1.7$ km; see heavy yellow band in Fig. 3 wheel) while 26% involved more extensive excursions (average diameter $>\,3.0$ km; heavy orange band in Fig. 3 wheel). Of this latter group, 8% were transverse-type trips (type 4, orange, i.e., heading to a destination and then returning with relative little deviation). Two examples of DARs in each category are shown in Fig. 3, along with the proportions of each type.

**Table 2:** The means and standard deviations of the four distance measures (all in kilometers) within the 7 categories of DARs (also see Fig. 3 and Additional file 1: Fig. B.4 in Appendix B)

DAR ID (color, %)	Net displ. (km)	Max. displ. (km)	Max. diam. (km)	Max. width (km)	Spatial description
1 (yellow, 27%)	0.11 ± 0.16	0.66 ± 0.35	0.79 ± 0.38	0.32 ± 0.15	Closed, very small local
2 (dark yellow, 28%)	0.10 ± 0.13	1.5 ± 0.5	1.7 ± 0.5	0.71 ± 0.23	Closed, local excursions
3 (light blue, 16%)	0.41 ± 0.58	2.1 ± 0.6	2.4 ± 0.6	1.2 ± 0.4	Partially open, small extensive excursions
4 (orange, 8%)	0.07 ± 0.07	3.4 ± 0.5	3.6 ± 0.5	0.70 ± 0.26	Closed, transverse excursions
5 (light red, 10%)	0.09 ± 0.14	3.0 ± 0.6	3.5 ± 0.6	1.9 ± 0.5	Closed, medium extensive excursions
6 (dark red, 8%)	0.13 ± 0.33	5.6 ± 1.7	6.1 ± 1.9	2.0 ± 1.3	Closed, large excursions
7 (blue, 3%)	4.1 ± 2.1	4.4 ± 2.2	4.8 ± 2.5	1.7 ± 1.2	Wide open, large extensive excursions

### Cluster distribution results

After having extracted the 7 clusters based on the chosen DAR measures, we proceeded by considering the starting points to observe DAR frequencies as well as by analysing the affects of seasonality, demographic factors and the landscape on the various DAR type distribution.

#### Spatial location clusters

**Fig. 4:**
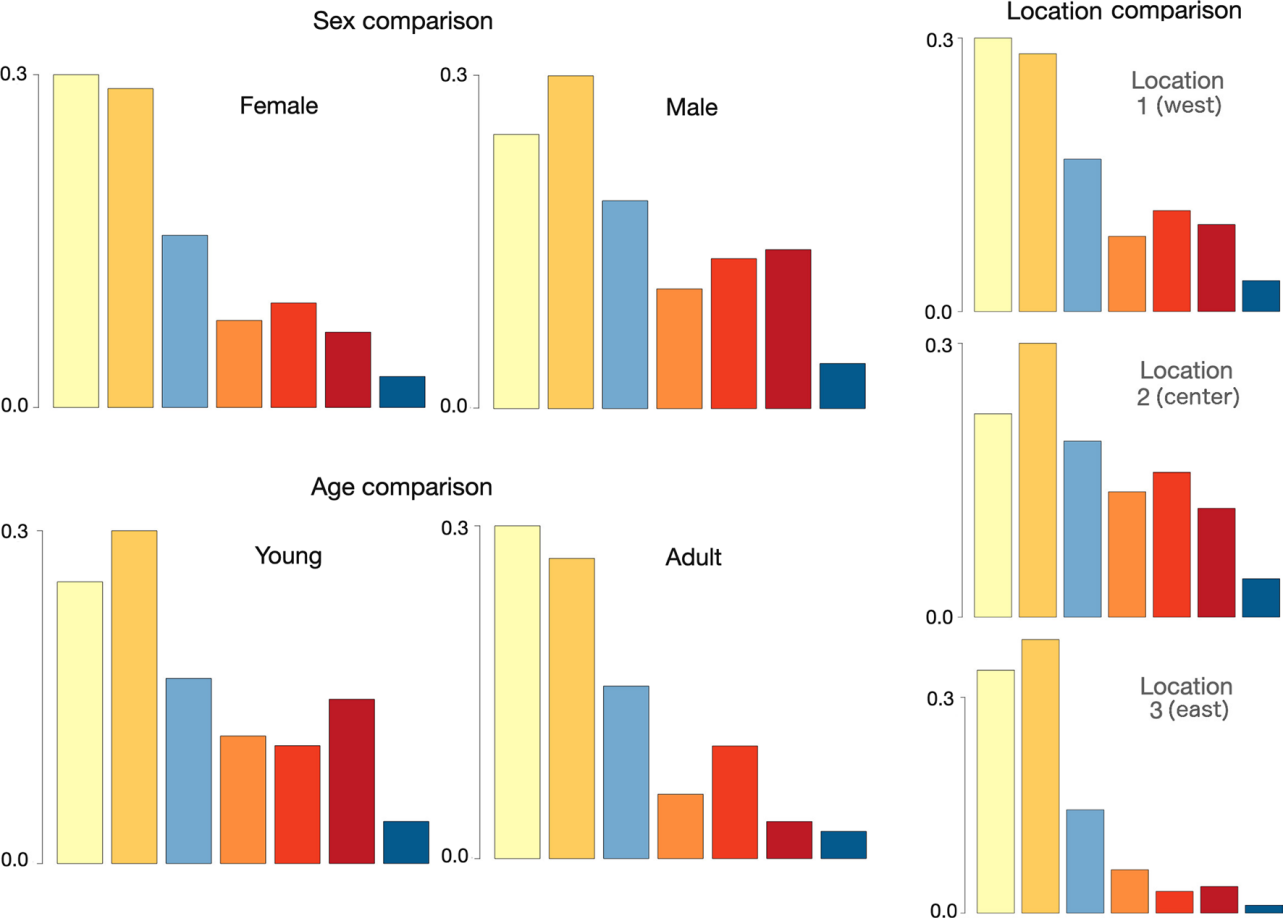
Bar plots of the frequency of DARs of each type (types 1–7 plotted from left to right using the same colors as referenced in Tables 1, 2, and used in Fig. 3) between sexes (top left two plots), ages (bottom left two plots) and DARs originating at three different locations (see text for discussion). Chi-squared contingency table analyses of these three comparisons (sex, age, location) are all highly significant ($$p<0.001)$$—see Additional file 1: Table A.3 in Appendix A for details

We used the (X,Y) coordinates of the start points of each DAR and then relied on a visual inspection (top left panel, Additional file 1: Fig. B.5) to select an appropriate number of clusters based on the “elbow” method (as described in the caption to Fig. 2A). In this way, we identified three start location categories of DARs, as illustrated in Additional file 1: Fig. B.5 (top right panel), which represent the western, central and eastern part of the studied area. In addition, the various areas differ in altitude, aridity and soil type. From Additional file 1: Fig. B.5 (bottom panel) we observe that the start point is quite consistent over time for each individual, showing consistent preference for particular areas. We use these spatial subdivision to group the DAR clusters and observe the effect of the initial location on their distribution (Fig. 4), as well as demographic factors, as explained in the next section.

#### Clustering subgroups by demographic and spatial attributes

The distribution of DAR types for different individuals is provided in Additional file 1: Table A.2 (Appendix A). These individuals can be organized into subgroups by, among other things, sex, age, and location (27 female, of which 11 young and 16 adults, and 17 males, of which 11 young and 6 adults). Bar plots of the number of DARs (i.e., charts of the distribution of DAR types when normalized to represent frequencies) of females compared with males, young ($\le 1$ year) compared with adults ($> 1$ year), and compared across locations 1 (west), 2 (center) and 3 (east), are illustrated in Fig. 4, with actual numbers provided in Additional file 1: Table A.3 (Appendix A). Note in the case of individuals maturing over time, the age of the individual is that assigned at the beginning of the data collection period, except for our GLMM analysis where we used the individual’s age at the time it executed the DAR under consideration. In a paper focusing on the ecology of the barn owl, it would be important to associate the appropriate developmental age of each individual at the time the particular DAR occurred. In Fig. 4 we show the aggregated distribution, while in the Additional file 1: Appendix B (Fig. B.6) we present the mean and standard deviation of the DAR frequency for each individual, organized by sex, age and location, presenting similar trends, as discussed in this section.

In the breeding season, males have greater home ranges than females [51]. This notable difference between sexes ($p<0.001$, Table A.3) formed as a result of the involvement of males in comparatively more medium and large excursions than females (light red and dark red bars; 24 vs 16%) and, conversely, females are involved in comparatively more local or small excursions than males (yellow and dark yellow bars; 58 vs 47 %). On the other hand, an obvious difference between young and adult individuals is that young embarked on a much greater proportion of large excursions then adults (dark red bar; 13 vs 4%) while adults embarked on a much greater proportion of local excursions than young individuals (yellow bar: 32 vs 23 %). These results suggest that adult females remain local during periods when they are involved in brood care and do happen to move—often though, while involved in brood care, females remain on the nest all night (and these events are not included in our analysis.

With regard to locations, a preponderance of trips at east locations were local or small (yellow and dark yellow; 72%), less so at west locations (54%), and even less so at central locations (45%). This consistent with the fact that the east location, being an agricultural grain field, is the richest of the three locations in food resources (highest density of rodents).

#### Temporal patterns

**Fig. 5:**
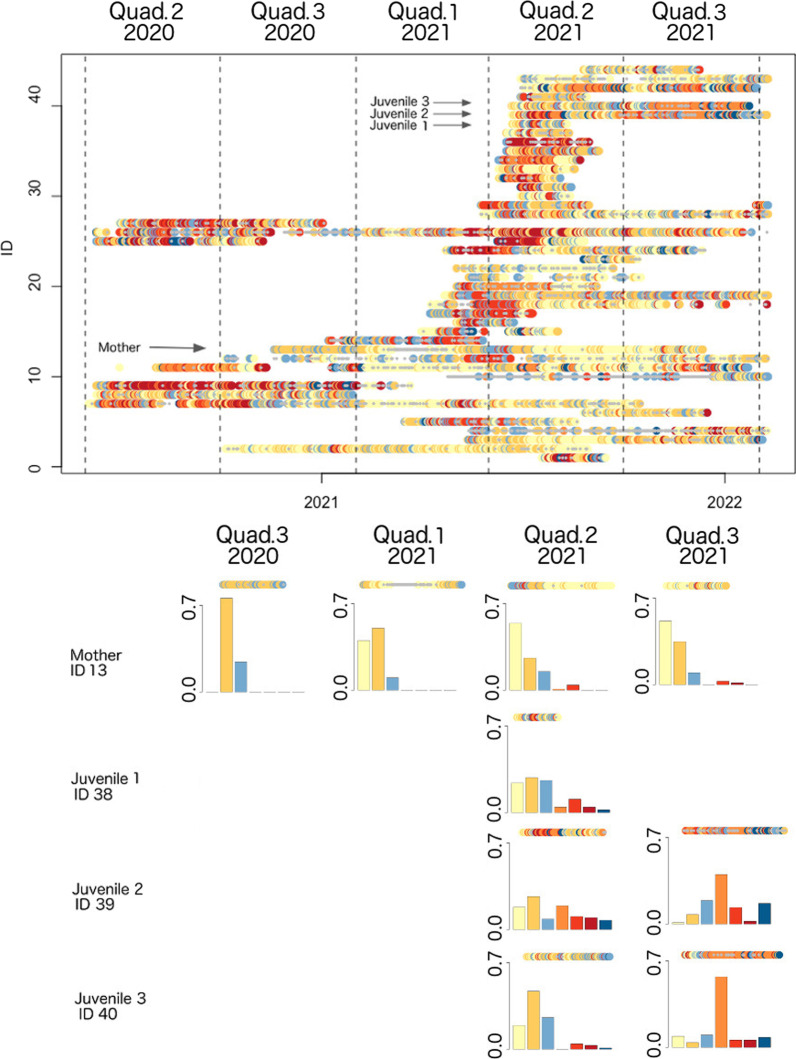
*Top panel*: Temporal distribution of the seven clusters, ordered by individual ID number (see Additional file 1: Table A.1), with dashed vertical lines dividing the different quadrimesters and small gray dots representing NA values in our measure extraction. For both panels, the color scheme is the same as that presented in Table 1. *Bottom panel*: Temporal distribution of the seven clusters. From the top: mother (ID 13) and her 3 fledglings (IDs 38, 39, and 40). We also show the temporal progression of the DAR types across quadrimesters, above each bar plot

We plotted the sequence of DARs, as they occurred for each of the individuals in the study (top panel in Fig. 5). We observe that the DAR distribution differs over time across individuals, and the different distributions can be explored to extract variations depending on age, sex, and choice of home area. We aggregated the individual DAR data for each individual bird and by quadrimesters as well, choosing 4 individuals from the same family (mother and her 3 young). Details of the family membership of individuals are known from regular monitoring of nest boxes. By evaluating the individual temporal distribution, we observe that certain DARs are more frequent during particular times of the year. For example (bottom panel in Fig. 5), during the 1st, 2nd and 3rd quadrimesters of 2021 (February–May 2021, June–September 2021, October 2021–January 2022) the female individual (mother) mostly shows closed local and small excursion DARs, which can connect with the behavior during and following the breeding period. In addition, the two young in the 3rd quadrimester of 2021 show a high frequency of type 4 DARs (closed, transverse excursion).

#### Landscape resources

**Fig. 6:**
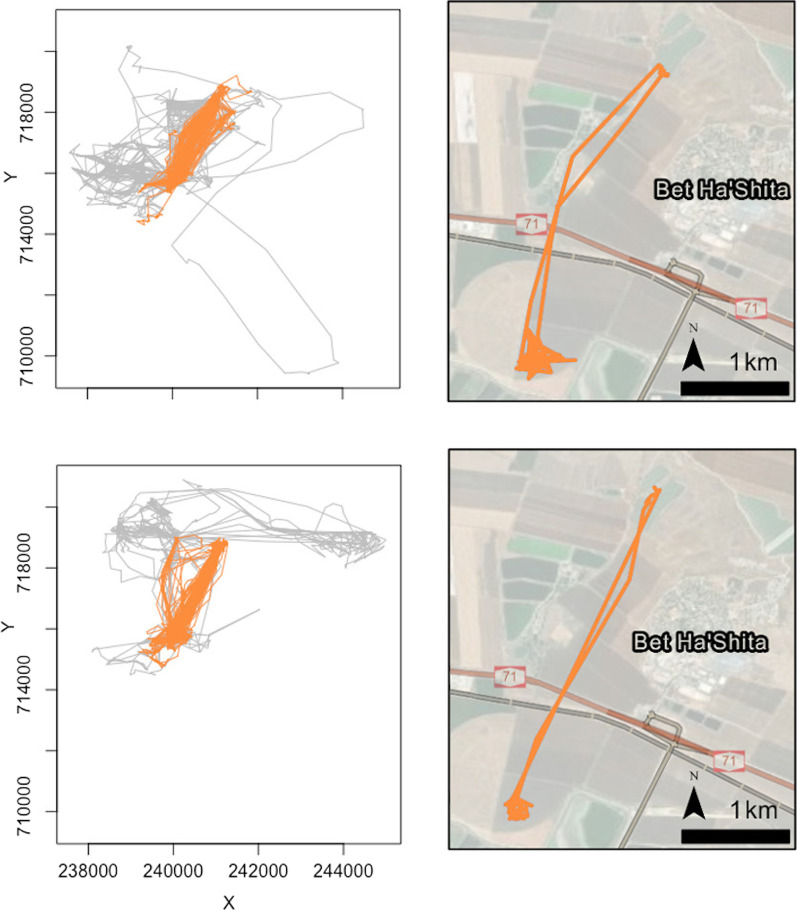
*Left panels*. Plots on the same scale of type 4 (orange) DARs for sibling fledglings (ID 39, top; ID 40 bottom)—these occurred over the same quadrimester (Oct 2021–Jan 2022) with other DARs from this quadrimester superimposed in gray. X and Y axes represent Israeli Transverse Mercator (ITM) coordinates. *Right panels.* Satellite image examples of single DARs (ID 39 on 2021-11-16, top; ID 40 on 2021-12-21 bottom)

By analysing the spatial locations of the closed, transverse excursion DAR (type 4, orange) performed by the 2 young individuals (highest frequency in bar plots in Fig. 5), we observe a very similar use of the landscape and resources, as shown in Fig. 6 (right panel). Note that for the two young we have a total of 36 and 57 DARs of type 4 for the considered quadrimester, over a different sets of days with 15 days in common.

In addition, we analyzed the DAR distributions and trajectories of a second family of four owls: mother, father, and two young individuals. For this family we were able to track both parents during the brood rearing period. The DAR distributions and trajectories of this family are presented in Additional file 1: Appendix B (Figs. B.7 and B.8).

### Generalized linear mixed model results

The detailed results of fitting a GLMM to the data—dependent variable was the square-root PC1, independent fixed binary variables were sex (male/female) and age (young/adult), random variables were RingID and date—are given in Additional file 1: Appendix C. The assumption that the square-root transformed PC1 is normally distributed appears to be reasonable from Q-Q and histogram plots of the residuals (Additional file 1: Fig. C.2B). For purposes of comparison, we also report the results for the same GLMM fit, except in this case the dependent variable was now the square-root of the DAR measure maximum displacement (Additional file 1: Fig. C.2B). The results of these two GLMM fits are almost identical (compare panels A and B in Additional file 1: Fig. C.2) apart from the fact that, as expected (because of its property in being the DAR-size composite variable that accounts for more of the variation than any other DAR-size variable), the PC1 fit provides marginally stronger statistical results. In the case of both GLMM fits, as was evident from our cluster results as well, DARs of young are significantly larger than those of adults at the highest level ($p<0.001$), while males have significantly larger DARs ($p<0.05$) than females. Adding the cross-product sex $\times$ age of the two variables to the GLMM did not improve the performance of the model sufficiently to justify its acceptance on information theoretic grounds (Additional file 1: Appendix C).

## Discussion

The primary purpose of this paper is to present a geometric method for categorizing diel activity routines (DARs) across days and across individuals in a population, and to demonstrate the utility of such categorization in providing insights into their behavior and movement ecology. Our approach may be best applied to groups of individuals for which relocation data support, at least, several points per hour. Since we had many more points than needed, we subsampled our 4–8 s interval data at 5 min intervals to save computation time in generating our measures with little observed loss of accuracy—e.g., compare tracks in Additional file 1: Fig. B.1 in Appendix B.

Of course, DAR categorization methods may use factors that differ and complement our geometric approach. Such approaches may be useful in uncovering area-related features (e.g., the amount of area searched in each diel cycle), or in identifying regions of the landscape where particular types of movement behavior occur (e.g., movement corridors or foraging areas [52]). We believe, however, that our geometric approach provides an appropriate first cut to characterizing segments at the DAR scale. The particular set of measures that we used in our study (net displacement, maximum displacement, maximum diameter, and maximum width) appear to have worked well in providing us with 7 meaningful types of DARs that we could then use to obtain ecologically useful information on the movement behavior of barn owl individuals. As we saw in our barn owl analysis, for example, the spatial results we obtained using a geometric categorization of DARs provides a daily-resolution lens on potential links between the movement and resource ecology of individuals. Our results also provide some insights into the variations in DAR ecology with respect to age and sex, as well the spectrum of personality types (behavioral syndromes; [22, 53]) that one may expect to encounter and how landscape and other environmental factors may alter this spectrum. Our results also allowed us to identify both similarities and differences among nest mates, as well as age-dependent seasonal response, spatial associations among DAR types, and the locations where these associations occurred.

According to the results that we presented in Fig. 2C, the values $k=2$ and $k=4$ are indicated as possible break-point values for selecting the number of clusters on which to conduct a comparative analysis. As we have mentioned, we decided to carry out our analysis using 7 clusters because of the greater diversity it offered to us in making the various comparisons across sex, age, and location, as illustrated in Figs. 4 and 5. We suggest, however, that four clusters may be a suitable alternative when the focus is on the coarse-grain movement patterns, or if the sampling rate is infrequent, though some understanding may be lost when the diversity of DAR movement patterns performed is reduced to four, and even more so to two.

Of course, we could have also carried out our comparisons using $k=$ 2 and 4. Such analyses are recommended when the focus is on the movement ecology of the population under consideration rather than on a methodological exposition, which is the purpose of the current paper. Additionally, because the main scope of this study is to showcase the relevance of our approach, we have limited our presentation to a few illustrative examples, as well as to the $k=7$ cluster selection. A more extensive ecological analysis of considerably more data than that reported here is analysed and discussed elsewhere [49].

The process of finding a useful DAR categorization scheme does not require a PCA to be undertaken. Further, as we mentioned, PCA is most useful when the number of variables involved is on the order of tens rather than a few to several. The PCA analysis that we undertook, however, was useful because it provided a method for generating a DAR-size composite variable (PC1, has the natural interpretation of size or “extent”) that explains more of the variation than any other linear combination (and, of course, with all weights positive) of the underlying measures used to characterize our DARs. In our case, our DAR extent variable, PC1, accounted for a remarkable 86.6% of the observed variation among DARs. Additionally, beyond the utility of our PCA identifying PC1, we note that 94.9% of the variation is captured by the first two principal components (PC1 and PC2—with the latter have the interpretation of DAR openness; Table 2), so that 2-D plots using PC1 and PC2 as their axes (such as Fig. 2D) contain almost all of the information extracted in our cluster analysis. Another advantage of our PCA pertains to our selection of PC1 as the dependent variable for our GLMM analysis of the effects of age and sex on DAR size. Although, using max displacement, as discussed below in the context of barn owl movement, provided better discriminative power, than max displacement (or any one of the geometric measures for that matter on its own).

We divide our ensuing discussion into three subsections. First, we discuss insights provided by the data on the movement patterns of the barn owls. Second, we discuss aspects of the current implementation of our method and overall approach. Third, we point out several ways forward, to obtain a deeper understanding of the nature and structure of the DARs of animals exhibiting strong day-to-day, seasonal and even lunar cycle variation in these routines.

### Barn owl diel movement patterns

With 44 individuals tracked over almost half a year on average, our focal barn owl data set is sufficiently general to obtain some useful insights into the movement behavior of individuals within our study population. We evaluated and compared the distribution DAR types with regard to sex, age and location/region. These comparisons nicely demonstrated some commonly known behavioral patterns regarding differences among sex and age, and individuals. In particular, our analysis reinforces results from other studies that adults move more locally compared to young [51]. Similarly, our results also suggest that males forage over larger distances that females. This is inline with recent results from another ATLAS-based study [39] and inline with the general notion that females move locally or very little while incubating their eggs. Individuals are expected to move less in areas of high resource availability, and indeed we find that that DAR distributions are strongly influenced by site location. Specifically, we found more local and closed small-excursion DARs in the eastern relatively high resource region than the other two locations where nest box densities where higher and resources abundances were lower.

Our clustering method also provided some novel insights not revealed by prior analyses of movement data; in particular, insights obtained from observed differences in DAR distributions among different family groups. Among the 4 family members included in Fig. 5 (bottom panel), the mother (individual 13) has a very different DAR distribution over time compared with each of her three offspring (all 4 identified by arrows in the top panel of Fig. 5). The predominance of closed local DARs (type 1, yellow) in the mother’s 1st quadrimester coincides with her known behavior during her egg incubation period. Further, because females remain nest bound during incubation [51], many of her DARs are recorded as NA during this quadrimester. Once the nestlings hatch, the mother is associated with two types of DARs: small-excursion DARs (type 2, dark yellow), which suggests flights to bring food to the nest (central place foraging); and some partially open, small-medium excursion DARs (type 3, light blue), which suggests flights to nearby feeding areas, presumably undertaken to avoid local resource depletion. After the fledgling period, we observe that the mother returns to performing closed local DARs, perhaps because she has no need to feed the fledglings anymore and so the local habitat area provides enough resources for her alone. Within the same family, we see a predominance of closed, transverse-excursion DARs (type 4, orange) for young individuals 2 and 3 in the third quadrimester of 2021 (bottom two rows of panels in Fig. 5). These two young individuals perform consistent and similar commuting patterns (roosting-foraging areas) across space and time (Fig. 6). In particular, the individuals moved between their roosting areas (banana plantation located in the upper right corner of the map provided in Fig. 6) and a rodent-rich alfalfa crop area (in the lower left corner of this map), where the individuals could find staple diet items [54, 55].

For the other family where we were able to track both parents during the brood rearing period, the DAR distributions and trajectories, presented in Additional file 1: Appendix B (Figs. B.7 and B.8), revealed the following. Their eggs hatched at the beginning of April, and the nestlings fledged and left the nest during the 2nd quadrimester. During this quadrimester, these young executed small, transverse excursions, visiting areas different from their parents, thereby establishing independent home ranges. The mother performed mostly closed, local DARs (type 1, yellow) and the father mostly closed, medium excursion (type 5, light red) [39]. In addition, we observed that during the 1st quadrimester the father showed a prevalence of partially open, small-medium excursion DARs (type 3, light blue). This supports the supposition that the father does not always roost in the nest during the incubation period [51], but spends some nights away from the nest.

Our two GLMM analyses in which PC1 and then the max displacement variable were regressed against the fixed binary variables of sex and age, and the random variables ringID and date, identified significant age and sex differences in DAR size: young males executed the most extensive and adult females the least extensive DARs. It is worth noting that both these analyses only considered non-zero DARs (i.e., the individual left its nest for some period of time during the day) because brooding females usually do not perform DARs while incubating eggs on their nests [49].

### Method applications

Any in depth study should consider appropriate measures for the DAR classification, as well as evaluate whether the initial number of clusters identified (7 in our case) is the most appropriate categorization scheme for addressing particular questions, in terms of the generality/specificity trade-offs discussed in the Introduction. In fact, the best number of clusters used to address particular questions may differ from question to question. In our case, the 7 groups we identified could be categorized rather succinctly into four size classes (local, and small, medium and large non-local excursions; see Fig. 3), and then in terms of whether larger excursions were closed, partially open or wide open (Table 1). The measures we used provided a geometric categorization of our set of DARs, where other types of categorization are possible, such as related to space use (amount of area searched, use of corridors, etc.).

The temporal patterns we obtained (as in Fig. 5) may be particularly germane to understanding the influence of lunar and solar cycles on the ecology of species. In addition, our approach can help identify individual behaviors. In particular, by summarizing temporal, spatial and DAR distribution patterns, we can evaluate behavior differences across individuals and therefore extract and quantify individual variations in behavior, an endeavor that has received growing attention over the last few years [22, 56].

An alternative to the approach we took, could use measures such as tortuosity [19, 57], but such measures are more relevant to canonical activity mode (CAM) than DAR categorizations. The reason is that different CAM segments of a single DAR will vary greatly with respect to tortuosity as individuals switch from a directed movement CAM (low tortuosity) to a forage or search CAM within a relatively small area (moderate to high tortuosity). One could also estimate average speed or distance travelled along CAM segments but this requires that relocation points are collected at a sufficiently high frequency and methods are sufficiently robust to be as scale independent as possible [21]. We also did not use 2-dimensional measures that are more suitable for characterizing the multi-day coverage of a collection of DARs [23] constituting a single LiMP than characterizing any single flight segment, unless the measure relates to the efficiency of a within patch search segment (i.e., a single search CAM).

In general, the geometry of clearly demarcated flight segments (i.e., segments with clear start and end points, book-ended by extended periods of rest)—such as our DARs—are most appropriately characterized using a set of scalar (1-dimensional) whole-path metrics (with respect to the DARs as a whole), such as maximum diameter, maximum width, net displacement, and maximum displacement. On the other hand, component segments—such as CAMs—require some type of behavioral change point analysis (BCPA) to separate them out, using various local type measures, such as tortuosity, average speed, and possibly area covered if the CAM is a within-patch search activity. Finally, multi-DAR segments, such as LiMPS or LiTs, are most appropriately characterized using a set of 2-dimensional measures relating to seasonal home-range size and overlaps between such home ranges in different seasons [23, 58], although 1-dimensional local metrics applied at an appropriate scale (likely at the hourly or subhourly rather than minute or subminute level) relating to total distance covered within seasons, or mean and variance of distances covered across days within different seasons may prove useful for categorizing LiMPs and LiTs.

### Next steps in the hierarchical segmentation of movement paths

Past studies on the spatial categorization and temporal patterning of DARs have involved relatively low frequency data (e.g., a couple of points per day to hourly or half-hourly relocation periods; [59–63]). As the first study to undertake a comprehensive categorization of the spatial structure and temporal patterning of DARs using minute resolution data, our focus has been on spatial categorization and temporal patterning methodology with only superficial links to landscape factors. Thus, our ability to drill deeply into links between movement and resource ecology of the barn owl in Israel has been limited. Future studies, no doubt will be able to combine our methodology with much more detailed landscape information to dig more deeply into the nexus among resource, movement, and other components of behavioral and feeding ecology than we have here.

Beyond extending an analysis of DARs to include various types of environmental factors, we are motivated in a movement ecology context to delve further into the segmentation of the DARs themselves into smaller definable units that provide a hierarchical framework for resolving movement trajectories [6]. In this framework, it has been proposed that DARs are themselves composed of a sequence of canonical activity modes (CAMs; [15]), which in turn are constructed from strings of statistical entities called meta fundamental movement elements (or metaFuMEs—see Glossary; [6, 7]). The criterion for identifying a particular kind of CAM is that it should stably classifiable (i.e., consistent and repeatable), and interpreted in terms some goal oriented activity [6], such as foraging for resources, heading towards a desired location, partaking in regenerative rest, or employing a sequence of risk avoidance related activities.

Methods for segmenting DARs into CAMs include the application of hidden Markov models (HMMs) [64] or other approaches falling under the rubric of behavioral change point analyses (BCPA) [65–67]. One or more of these BCPA methods can be applied to the problem of segmenting DARs into biologically meaningful subunits, although how we do this can vary greatly with regard to organization of DARs into sets of data for independent analyses.

In the same way that BCPA methods can be used to segment DARs into CAMs, so CAMs can be segmented either in sequences of shorter duration CAMs (e.g., foraging bouts are segmented into moving between foraging points and harvesting resources around foraging points) or short duration CAMs themselves can be segmented into metaFuMEs, as discussed in [7]. Further, once several different sets of metaFuMEs have been identified, they can be used, also as discussed in [7], to simulate CAMs and DARs using metaFuME statistics and Markov matrices that control the transition probabilities of switches among metaFuME types and DARs. These simulations, however, are based solely on local information pertaining to step-size and turning angle distributions that defined particular metaFuME types, and Markov transition probabilities that switch among metaFuME types and particular CAM modes possible using time-of-day or location landscape information.

Realistic simulation of DARs, as a function of landscape and other environmental factors (including time and length of day), will generally require non-local information (i.e., beyond step-size and turning angle distributions). This information includes the location of desired destinations, stored in an individual’s memory and approached through navigation that may involve landscape markers or celestial objects. It is only through knowing the relationship of CAM movement sequences to landscape factors and movement motivated by non local information that the movement of animals at the DAR scale can be used to evaluate the response of individuals to changes in the landscape (as happen as a consequence of global warming in human land use activities).

## Conclusion

The methods we present here for categorizing the geometry of DARs are part of a broader program that involves gathering information at the diel scale to build models able to predict the ecological movement response of individuals to global change. These models are based on identifying sub-diel CAM segments, using behavioral change point analysis, and then associating these segments with particular environmental conditions and landscape structures [6]. These associations may then allow us to anticipate how DAR types and distributions will respond to changes in the landscape and environment. Once these models have been used to obtain a set of DARs adapted to these changes, they can then be strung together to predict how the LiMPS of individuals might respond to the same set of changes. This type of hierarchical, multiscale approach to constructing predictive movement models is likely to be much more flexible than single scale approaches in capturing the response of individuals to landscape and environmental change.

As we have seen with our barn owl study, the landscape structure of locations is key to influencing the frequency and distribution of DARs for individuals of particular ages, sex or behavioral types [49]. Thus a prediction of what sequences of DARs individuals will generate over a specific season in a particular location is very much dependent on the landscape changes that take place at that location over time. In particular, knowing how individuals will alter the structure of their DARs in response to landscape changes, and how these DARs are likely to be strung together into LiMPs (e.g., seasonal ranging, dispersal, migration) of the individual’s LiT [4] will provide the kind of information needed to mitigate the effects of global change on the movement ecology of individuals and, hence, the resource ecology of populations.

Currently, most movement models are fundamentally single scale. For example, one genre is based on fitting stochastic walks to movement trajectories: e.g., basic random walks (i.e., Wiener process [68]), linear- and angular-velocity-biased random walks (e.g., Ornstein-Uhlenbeck process [69], location-salient random walks imagined as movements in a force field [70]), or movements dependent on the local landscapes [71], as well as time itself [72]. Other approaches involve using rule-based simulations to move individuals over rasterized landscapes [73, 74]. Although these single scale models may produce realistic tracks at the canonical activity mode (CAM) scale, they will fail to produce realistic tracks at the DAR scale, unless they incorporate non-local information when formulating rules on where and how to move [7]. The challenge remains, however, on how reliably we can string CAMs together to produce an array of DARs that match the distributional features of the DAR types identified using our approach and also capture how these distributions will adapt to environmental and landscape change. Only time can tell how well we can meet this challenge.

### Supplementary Information

**Additional file 1**. Additional tables, figures and results from our generalized linear mixed models analyses.

## Data Availability

The supplementary online file contains the following appendices: Appendix A, Additional Tables and Results; Appendix B, Additional Figures; Appendix C, GLMM Analysis. The datasets and the R code supporting the conclusions of this article are available in the Github repository https://github.com/LudovicaLV/DAR_project.

## Glossary

### Acronyms

An expansion of the various acronyms used in the text are provided here in alphabetical order along with a very brief definition/explanation of their meaning.

ATLAS: Advanced Tracking and Localization of Animals in real-life Systems [47].
BPCA: Behavioral (also Statistical) Change Point Analysis. This refers to a group of methods used to determine how the statistics of an independent biological variable (e.g., its mean, variance, autocorrelation, or rate of change in slope or curvature), switches at threshold points with changes in a dependent variable (e.g., space or time; [65, 75, 76]).
CAM: Canonical Activity Mode. This is a stably classifiable subdiel behavioral mode (pattern of movement) such as a foraging bout, resting period, or purposeful heading (i.e., traveling) to a distant target location.
DAR: Diel Activity Routine. This is a stably classifiable 24-hour sequence of CAMs that occur at characteristic frequencies and times of the day. We note that the start and end of a DAR cycle may vary for diurnal, nocturnal and crepuscular species.
FuME: Fundamental Movement Elements (pronounced “fume” and same as FME defined in [15]). This is a relatively rapid, highly repeatable, stereotypic set of body movements that forms the basis of the locomotory capacity of an individual (e.g., a walk step, a running step, a wing flap, a jump, etc.).
GLMM: Generalized Linear Mixed Models. This is a statistical approach to identifying the extent to which different fixed variables, in our case age and sex, determine the value of a dependent variable, such as PC1 or max displacement, in the presence of random effects variables that account for repeated measures or differences in times of the season when the data were collected.
HMM: Hidden Markov Models. A method for associating an unobservable state (e.g., a behavioral state) with an observable process (e.g., a time series of locations) [64].
LiMP: Lifetime Movement Phase. This is a path segment that typically reflects a life-history relevant movement behavior such as dispersal (episodic), migration (periodic), or other periodic behaviors at a greater-than-diel scale.
LiT: Lifetime Track. This is the total movement path of an individual from its birth to its death.
MetaFuME: A correlated stereotypical or characteristic sequence of FuMEs of fixed duration equal to the time between consecutive relocation points (only applicable to relatively high frequency data: ideally, metaFuMEs should contain no more than several tens of FuMES).
PCA: Principal components analysis [31].
PC*i*: Principal component *i*, $i=1,2,3,4.$

### Terms

Extent: in the context of our DARs, this could very loosely be thought of as the size, magnitude or amount of area encapsulated by a DAR.
Elongation: in the context of our DARs, this refers to DARs which have a large maximum diameter and small maximum width.
Maximum diameter [MYAMP Diameter line]: in the context of our DARs, this refers to the greatest distance between any two points on the DAR, where the line segment joining these two points is referred to as the diameter line.
Maximum displacement: in the context of our DARs, this refers to the distance from the starting point to the most distant point on the DAR.
Maximum width: in the context of our DARs, this refers to the sum of the maximum distances of the trajectory points on either side of the diameter line.
Net displacement: in the context of our DARs, this is the distance between the locations of the start and finish of each DAR.
Openness: if the net displacement is of the order of several meters or less, then the DAR is closed (i.e., the individual returned essentially to its starting location), otherwise the DAR is open, where if net displacement is close to maximum displacement, the DAR can be thought of as maximally open or a one-way translocation/journey.

## References

[1] Kays R, Crofoot MC, Jetz W, Wikelski M (2015). Terrestrial animal tracking as an eye on life and planet. Science.

[2] Marvin DC, Koh LP, Lynam AJ, Wich S, Davies AB, Krishnamurthy R, Stokes E, Starkey R, Asner GP (2016). Integrating technologies for scalable ecology and conservation. Glob Ecol Conserv.

[3] Nathan R, Monk CT, Arlinghaus R, Adam T, Alós J, Assaf M, Baktoft H, Beardsworth CE, Bertram MG, Bijleveld AI (2022). Big-data approaches lead to an increased understanding of the ecology of animal movement. Science.

[4] Nathan R, Getz WM, Revilla E, Holyoak M, Kadmon R, Saltz D, Smouse PE (2008). A movement ecology paradigm for unifying organismal movement research. Proc Natl Acad Sci.

[5] Spiegel O, Harel R, Centeno-Cuadros A, Hatzofe O, Getz WM, Nathan R (2015). Moving beyond curve fitting: using complementary data to assess alternative explanations for long movements of three vulture species. Am Nat.

[6] Getz WM (2022). A hierarchical framework for segmenting movement paths. Ecol Process.

[7] Getz WM, Luisa Vissat L, Salter R. Simulation and analysis of animal movement paths using numerus model builder. In: 2020 spring simulation conference (SpringSim). IEEE, pp 1–12 (2020)

[8] Johnson DS, London JM, Lea M-A, Durban JW (2008). Continuous-time correlated random walk model for animal telemetry data. Ecology.

[9] Ahearn SC, Dodge S, Simcharoen A, Xavier G, Smith JL (2017). A context-sensitive correlated random walk: a new simulation model for movement. Int J Geogr Inf Sci.

[10] Breed GA, Golson EA, Tinker MT (2017). Predicting animal home-range structure and transitions using a multistate Ornstein–Uhlenbeck biased random walk. Ecology.

[11] Häfker NS, Tessmar-Raible K (2020). Rhythms of behavior: are the times changin’?. Curr Opin Neurobiol.

[12] Bloch G, Barnes BM, Gerkema MP, Helm B (2013). Animal activity around the clock with no overt circadian rhythms: patterns, mechanisms and adaptive value. Proc R Soc B Biol Sci.

[13] Moran D, Softley R, Warrant EJ (2014). Eyeless Mexican cavefish save energy by eliminating the circadian rhythm in metabolism. PLoS ONE.

[14] Beale AD, Whitmore D, Moran D (2016). Life in a dark biosphere: a review of circadian physiology in “arrhythmic” environments. J Comp Physiol B.

[15] Getz WM, Saltz D (2008). A framework for generating and analyzing movement paths on ecological landscapes. Proc Natl Acad Sci.

[16] Wittemyer G, Polansky L, Douglas-Hamilton I, Getz WM. Disentangling the effects of forage, social rank, and risk on movement autocorrelation of elephants using Fourier and wavelet analyses. In: Proceedings of the national academy of sciences. 2008;0801744105. 10.1073/pnas.0801744105 PMC2614723 19060207

[17] Yackulic CB, Blake S, Deem S, Kock M, Uriarte M (2011). One size does not fit all: flexible models are required to understand animal movement across scales. J Anim Ecol.

[18] Goossens S, Wybouw N, Van Leeuwen T, Bonte D (2020). The physiology of movement. Mov Ecol.

[19] Benhamou S (2004). How to reliably estimate the tortuosity of an animal’s path: straightness, sinuosity, or fractal dimension?. J Theor Biol.

[20] Bradshaw CJ, Sims DW, Hays GC (2007). Measurement error causes scale-dependent threshold erosion of biological signals in animal movement data. Ecol Appl.

[21] Noonan MJ, Tucker MA, Fleming CH, Akre TS, Alberts SC, Ali AH, Altmann J, Antunes PC, Belant JL, Beyer D (2019). A comprehensive analysis of autocorrelation and bias in home range estimation. Ecol Monogr.

[22] Sih A, Mathot KJ, Moiron M, Montiglio P-O, Wolf M, Dingemanse NJ (2015). Animal personality and state-behaviour feedbacks: a review and guide for empiricists. Trends Ecol Evol.

[23] Abrahms B, Seidel DP, Dougherty E, Hazen EL, Bograd SJ, Wilson AM, McNutt JW, Costa DP, Blake S, Brashares JS (2017). Suite of simple metrics reveals common movement syndromes across vertebrate taxa. Mov Ecol.

[24] Codling E, Hill N (2005). Sampling rate effects on measurements of correlated and biased random walks. J Theor Biol.

[25] Codling EA, Plank MJ (2011). Turn designation, sampling rate and the misidentification of power laws in movement path data using maximum likelihood estimates. Theor Ecol.

[26] Seidel DP, Linklater WL, Kilian W, du Preez P, Getz WM (2019). Mesoscale movement and recursion behaviors of Namibian black rhinos. Mov Ecol.

[27] Saxena A, Prasad M, Gupta A, Bharill N, Patel OP, Tiwari A, Er MJ, Ding W, Lin C-T (2017). A review of clustering techniques and developments. Neurocomputing.

[28] Murtagh F, Contreras P (2012). Algorithms for hierarchical clustering: an overview. Wiley Interdiscip Rev Data Min Knowl Discov.

[29] Tarca AL, Carey VJ, Chen X-W, Romero R, Drăghici S (2007). Machine learning and its applications to biology. PLoS Comput Biol.

[30] Valletta JJ, Torney C, Kings M, Thornton A, Madden J (2017). Applications of machine learning in animal behaviour studies. Anim Behav.

[31] Abdi H, Williams LJ (2010). Principal component analysis. Wiley Interdiscip Rev Comput Stat.

[32] Jolliffe IT, Cadima J (2016). Principal component analysis: a review and recent developments. Philos Trans R Soc A Math Phys Eng Sci.

[33] Getz WM, Page RE (1991). Chemosensory kin-communication systems and kin recognition in honey bees. Ethology.

[34] Bradshaw WE, Holzapfel CM (2007). Evolution of animal photoperiodism. Annu Rev Ecol Evol Syst.

[35] Martin LB, Weil ZM, Nelson RJ (2008). Seasonal changes in vertebrate immune activity: mediation by physiological trade-offs. Philos Trans R Soc B Biol Sci.

[36] Evens R, Kowalczyk C, Norevik G, Ulenaers E, Davaasuren B, Bayargur S, Artois T, Åkesson S, Hedenström A, Liechti F (2020). Lunar synchronization of daily activity patterns in a crepuscular avian insectivore. Ecol Evol.

[37] Polansky L, Wittemyer G, Cross PC, Tambling CJ, Getz WM (2010). From moonlight to movement and synchronized randomness: Fourier and wavelet analyses of animal location time series data. Ecology.

[38] Pitera A, Branch C, Bridge E, Pravosudov V (2018). Daily foraging routines in food-caching mountain chickadees are associated with variation in environmental harshness. Anim Behav.

[39] Corl A, Charter M, Rozman G, Toledo S, Turjeman S, Kamath PL, Getz WM, Nathan R, Bowie RC (2020). Movement ecology and sex are linked to barn owl microbial community composition. Mol Ecol.

[40] Weiser AW, Orchan Y, Nathan R, Charter M, Weiss AJ, Toledo S. Characterizing the accuracy of a self-synchronized reverse-GPS wildlife localization system. In: 2016 15th ACM/IEEE international conference on information processing in sensor networks (IPSN). IEEE, pp. 1–12 (2016).

[41] Orlowski J, Harmening W, Wagner H (2012). Night vision in barn owls: visual acuity and contrast sensitivity under dark adaptation. J Vis.

[42] Espíndola-Hernández P, Mueller JC, Carrete M, Boerno S, Kempenaers B (2020). Genomic evidence for sensorial adaptations to a nocturnal predatory lifestyle in owls. Genome Biol Evol.

[43] O’Farrell S, Sanchirico JN, Spiegel O, Depalle M, Haynie AC, Murawski SA, Perruso L, Strelcheck A (2019). Disturbance modifies payoffs in the explore-exploit trade-off. Nat Commun.

[44] Meyrom K, Motro Y, Leshem Y, Aviel S, Izhaki I, Argyle F, Charter M (2009). Nest-box use by the Barn Owl Tyto alba in a biological pest control program in the Beit She’an valley, Israel. Ardea.

[45] Toledo S, Shohami D, Schiffner I, Lourie E, Orchan Y, Bartan Y, Nathan R (2020). Cognitive map-based navigation in wild bats revealed by a new high-throughput tracking system. Science.

[46] Toledo S, Mendel S, Levi A, Vortman Y, Ullmann W, Scherer L-R, Pufelski J, van Maarseveen F, Denissen B, Bijleveld A, Orchan Y, Bartan Y, Margalit S, Talmon I, Nathan R. Vildehaye: a family of versatile, widely-applicable, and field-proven lightweight wildlife tracking and sensing tags. In: Proceedings of the ACM/IEEE international conference on information processing in sensor networks (IPSN) (2022). 10.1111/2041-210X.13913

[47] Beardsworth C, Gobbens E, van Maarseveen F, Denissen B, Dekinga A, Nathan R, Toledo S, Bijleveld A (2022). Validating ATLAS: a regional-scale, high-throughput tracking system. Methods Ecol Evol.

[48] Gupte PR, Beardsworth CE, Spiegel O, Lourie E, Toledo S, Nathan R, Bijleveld AI (2022). A guide to pre-processing high-throughput animal tracking data. J Anim Ecol.

[49] Cain S, Solomon T, Leshem Y, Toledo S, Eitem A, Roulin A, Spiegel O. Movement predictability of individual barn owls facilitates estimation of home range size and survival. Mov Ecol In press. 2023. 10.1186/s40462-022-00366-x PMC9906850 36750910

[50] Murtagh F, Legendre P (2014). Ward’s hierarchical agglomerative clustering method: which algorithms implement Ward’s criterion?. J Classif.

[51] Roulin A (2020). Barn owls: evolution and ecology.

[52] Bastille-Rousseau G, Wittemyer G (2021). Characterizing the landscape of movement to identify critical wildlife habitat and corridors. Conserv Biol.

[53] Spiegel O, Leu ST, Sih A, Godfrey SS, Bull CM (2015). When the going gets tough: behavioural type-dependent space use in the sleepy lizard changes as the season dries. Proc R Soc B Biol Sci.

[54] Bose M, Guidali F (2001). Seasonal and geographic differences in the diet of the barn owl in an agro-ecosystem in northern Italy. J Raptor Res.

[55] Viganò M, Ancillotto L, Agnelli P, Ficetola GF, Mori E (2020). Frequency of occurrence and ingested biomass of different prey of the Barn Owl Tyto alba in an island ecosystem. Birds.

[56] Spiegel O, Leu ST, Bull CM, Sih A (2017). What’s your move? Movement as a link between personality and spatial dynamics in animal populations. Ecol Lett.

[57] Avgar T, Potts JR, Lewis MA, Boyce MS (2016). Integrated step selection analysis: bridging the gap between resource selection and animal movement. Methods Ecol Evol.

[58] Payne E, Spiegel O, Sinn D, Leu S, Gardner M, Godfrey S, Wohlfeil C, Sih A (2022). Intrinsic traits, social context, and local environment shape home range size and fidelity of sleepy lizards. Ecol Monogr.

[59] Van Dyck H, Baguette M (2005). Dispersal behaviour in fragmented landscapes: routine or special movements?. Basic Appl Ecol.

[60] Klaassen RH, Strandberg R, Hake M, Alerstam T (2008). Flexibility in daily travel routines causes regional variation in bird migration speed. Behav Ecol Sociobiol.

[61] Owen-Smith N, Goodall V, Fatti P (2012). Applying mixture models to derive activity states of large herbivores from movement rates obtained using GPS telemetry. Wildl Res.

[62] Morelle K, Podgórski T, Prévot C, Keuling O, Lehaire F, Lejeune P (2015). Towards understanding wild boar sus scrofa movement: a synthetic movement ecology approach. Mammal Rev.

[63] Owen-Smith N, Hopcraft G, Morrison T, Chamaillé-Jammes S, Hetem R, Bennitt E, Van Langevelde F (2020). Movement ecology of large herbivores in African savannas: current knowledge and gaps. Mammal Rev.

[64] Zucchini W, MacDonald IL (2009). Hidden Markov models for time series: an introduction using R.

[65] Gurarie E, Andrews RD, Laidre KL (2009). A novel method for identifying behavioural changes in animal movement data. Ecol Lett.

[66] Gurarie E, Bracis C, Delgado M, Meckley TD, Kojola I, Wagner CM (2016). What is the animal doing? Tools for exploring behavioural structure in animal movements. J Anim Ecol.

[67] Owen-Smith N, Martin J (2015). Identifying space use at foraging arena scale within the home ranges of large herbivores. PLoS ONE.

[68] McClintock BT, Johnson DS, Hooten MB, Ver Hoef JM, Morales JM (2014). When to be discrete: the importance of time formulation in understanding animal movement. Mov Ecol.

[69] Gurarie E, Fleming CH, Fagan WF, Laidre KL, Hernández-Pliego J, Ovaskainen O (2017). Correlated velocity models as a fundamental unit of animal movement: synthesis and applications. Mov Ecol.

[70] Magdziarz M, Metzler R, Szczotka W, Zebrowski P (2012). Correlated continuous-time random walks in external force fields. Phys Rev E.

[71] Harris KJ, Blackwell PG (2013). Flexible continuous-time modelling for heterogeneous animal movement. Ecol Model.

[72] Langrock R, Hopcraft JGC, Blackwell PG, Goodall V, King R, Niu M, Patterson TA, Pedersen MW, Skarin A, Schick RS (2014). Modelling group dynamic animal movement. Methods Ecol Evol.

[73] Getz WM, Salter R, Lyons AJ, Sippl-Swezey N (2015). Panmictic and clonal evolution on a single patchy resource produces polymorphic foraging guilds. PLoS ONE.

[74] del Mar Delgado M, Miranda M, Alvarez SJ, Gurarie E, Fagan WF, Penteriani V, di Virgilio A, Morales JM (2018). The importance of individual variation in the dynamics of animal collective movements. Philos Trans R Soc B Biol Sci.

[75] Morales JM, Haydon DT, Frair J, Holsinger KE, Fryxell JM (2004). Extracting more out of relocation data: building movement models as mixtures of random walks. Ecology.

[76] Chen J, Gupta AK (2011). Parametric statistical change point analysis: with applications to genetics, medicine, and finance.

