# Supplementary material for: Categorizing the geometry of animal diel movement patterns with examples from high-resolution barn owl tracking
Source: Mov Ecol. 2023 Mar 21;11:15. doi: 10.1186/s40462-023-00367-4 (PMC10029274; doi:10.1186/s40462-023-00367-4)
Supplement: Supplementary file 1 — Additional file 1. Additional tables, figures and results from our generalized linear mixed models analyses. [file 40462_2023_367_MOESM1_ESM.pdf]

Appendices

Appendix A: Additional Tables and Results

Tables

Table A.1 ID number and technical identification, collection dates and number of considered DARs (ncD) for each individual in the study

#	ID	Collection dates	ncD	#	ID	Collection dates	ncD
1	GG18092	2021-07-19 → 2021-09-15	54	23	GG39226	2021-08-26 → 2021-10-16	36
2	GG19629	2020-10-06 → 2021-08-30	300	24	GG39262	2021-04-22 → 2021-12-14	190
3	GG26748	2021-05-13 → 2022-02-05	243	25	GG39394	2020-06-11 → 2021-08-28	207
4	GG29111	2021-05-13 → 2022-02-09	84	26	GG39395	2020-06-11 → 2022-02-08	401
5	GG30813	2021-03-16 → 2021-08-20	120	27	GG39406	2020-07-02 → 2021-01-03	159
6	GG36056	2021-08-26 → 2021-12-16	91	28	GG41258	2021-05-25 → 2022-02-09	199
7	GG37019	2020-06-04 → 2021-10-18	351	29	GG41259	2021-05-25 → 2022-02-09	103
8	GG37049	2020-06-11 → 2021-01-29	203	30	GG41260	2021-06-30 → 2021-08-14	35
9	GG37055	2020-06-11 → 2021-03-23	198	31	GG41269	2021-06-30 → 2021-08-16	46
10	GG37106	2021-04-25 → 2022-02-09	79	32	GG41283	2021-06-10 → 2021-08-05	50
11	GG37113	2020-07-02 → 2022-01-22	408	33	GG41284	2021-06-10 → 2021-08-19	61
12	GG37128	2020-10-06 → 2022-02-09	231	34	GG41285	2021-06-10 → 2021-08-23	68
13	GG37131	2020-11-19 → 2021-12-12	332	35	GG41301	2021-06-17 → 2021-10-10	82
14	GG37133	2021-01-07 → 2021-05-27	97	36	GG41303	2021-06-17 → 2021-09-01	45
15	GG37136	2021-04-01 → 2021-08-03	83	37	GG41305	2021-06-17 → 2021-08-15	44
16	GG37138	2021-05-03 → 2021-06-25	43	38	GG41309	2021-06-22 → 2021-08-12	46
17	GG37139	2021-04-09 → 2021-07-12	78	39	GG41310	2021-06-22 → 2022-02-09	188
18	GG37140	2021-04-09 → 2022-02-09	193	40	GG41312	2021-06-22 → 2022-01-23	194
19	GG37141	2021-04-22 → 2022-02-09	251	41	GG41336	2021-06-30 → 2021-08-29	42
20	GG37163	2021-05-03 → 2021-09-08	82	42	GG41339	2021-06-30 → 2022-01-30	201
21	GG37172	2021-05-13 → 2021-10-17	48	43	GG41340	2021-06-30 → 2022-02-09	117
22	GG39117	2021-05-03 → 2021-09-23	69	44	GG53006	2021-08-26 → 2021-12-09	78

Correlation Matrix

The following is an upper diagonal matrix of the correlation coefficients between the extracted DAR measures:

$$M = \begin{array}{c} \text{Net displ.} \quad \text{Max. displ.} \quad \text{Max. diam.} \quad \text{Max. width} \\ \begin{pmatrix} 1.00 & 0.32 & 0.33 & 0.22 \\ & 1.00 & 0.98 & 0.66 \\ & & 1.00 & 0.71 \\ & & & 1.00 \end{pmatrix} \end{array} \tag{A.1}$$

Table A.2 The number of DARs of each type (see Tables 1 and 2 in main text) for each individual listed in Table A.1.

#	ID	1	2	3	4	5	6	7
1	GG18092	14	7	5	2	8	10	8
2	GG19629	152	116	22	5	2	3	0
3	GG26748	94	98	30	8	8	3	2
4	GG29111	14	32	16	2	2	16	2
5	GG30813	26	29	31	6	24	2	2
6	GG36056	28	49	11	0	1	2	0
7	GG37019	108	90	51	31	33	36	2
8	GG37049	59	75	39	5	19	6	0
9	GG37055	12	29	18	10	26	91	12
10	GG37106	15	18	35	2	6	1	2
11	GG37113	173	56	23	80	38	13	25
12	GG37128	103	53	45	1	25	4	0
13	GG37131	130	144	49	1	7	1	0
14	GG37133	0	12	31	10	29	6	9
15	GG37136	33	15	13	1	9	5	7
16	GG37138	5	10	17	0	6	5	0
17	GG37139	3	13	24	0	32	6	0
18	GG37140	84	42	17	4	39	6	1
19	GG37141	20	93	84	16	38	0	0
20	GG37163	19	12	6	34	8	3	0
21	GG37172	19	13	15	0	1	0	0
22	GG39117	48	13	8	0	0	0	0
23	GG39226	8	19	4	0	1	0	4
24	GG39262	48	51	25	8	24	22	12
25	GG39394	39	44	31	2	16	56	19
26	GG39395	52	128	70	24	46	78	3
27	GG39406	19	39	27	6	29	39	0
28	GG41258	71	54	26	8	20	8	12
29	GG41259	3	44	23	0	31	2	0
30	GG41260	20	4	6	0	3	2	0
31	GG41269	3	17	10	2	6	7	1
32	GG41283	6	14	2	11	6	9	2
33	GG41284	38	13	2	3	2	3	0
34	GG41285	14	3	4	30	9	8	0
35	GG41301	7	31	16	12	6	10	0
36	GG41303	0	0	1	8	1	35	0
37	GG41305	11	21	5	0	5	2	0
38	GG41309	11	13	12	2	5	2	1
39	GG41310	18	35	26	54	22	11	22
40	GG41312	27	48	34	57	10	9	9
41	GG41336	1	27	7	1	3	1	2
42	GG41339	37	26	19	68	18	5	28
43	GG41340	71	27	17	0	2	0	0
44	GG53006	17	42	15	0	1	3	0

Table A.3 Distribution of the number of DARs in the 7 different categories at locations 1-3 (west, center and east), females, males, young ($\leq$ 1yr) and adult ($>$ 1yr) and the significance of the comparison of these distributions across groups.

ID DAR	No. DARs	Loc. 1	Loc. 2	Loc. 3	Female	Male	Young	Adult
1	1680	1211	222	247	1254	426	729	951
2	1719	1142	299	278	1202	517	861	858
3	972	675	192	105	649	323	479	493
4	514	333	137	44	328	186	330	184
5	627	447	158	22	394	233	305	322
6	531	385	119	27	284	247	425	106
7	187	137	42	8	117	70	109	78
**Significance**		$\chi^2 = 184.38,\ P < 0.001$			$\chi^2 = 103.16,\ P < 0.001$		$\chi^2 = 258.94,\ P < 0.001$	

4 **Appendix B: Additional Figures**

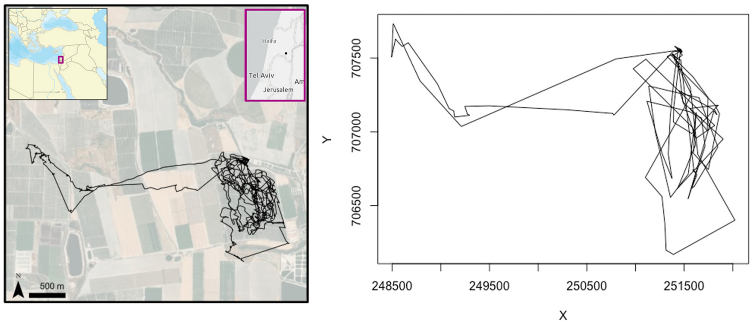

Figure B.1 A DAR trajectory example is plotted (left panel), as recorded within our Harod valley study area located in northern Israel (right inset). To reduce computational time, we computed the metrics from a 5-min subsampling (right panel) of the much higher frequency trajectory (left panel). We observe little loss of accuracy among collected and subsampled data (also see Fig. B.3).

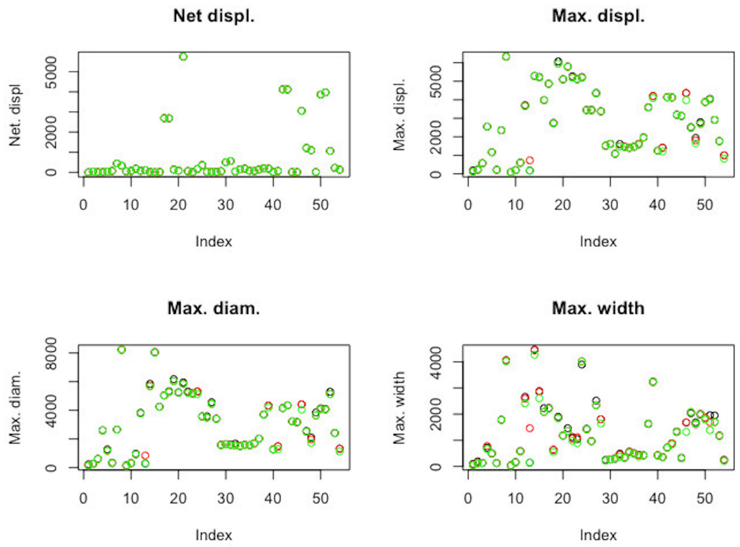

Figure B.2 The chosen 4 metrics calculated for individual ID 1, for resolution 2-min (black), 5-min (green) and 10-min (red). Note that the calculation of net displacement was independent of the resolution, and therefore the result was the same for each scenario.

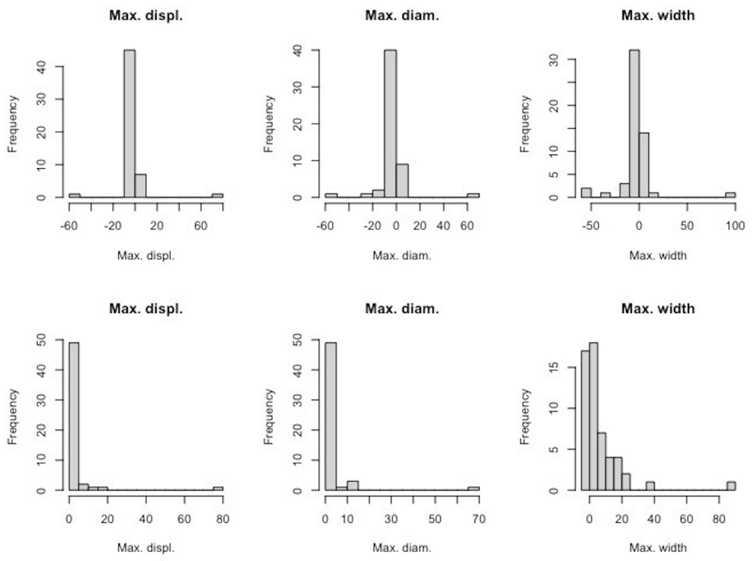

Figure B.3 Histograms of the relative change ρ for max.displ, max.diam. and max.width between 5-min resolution calculation and 2-min (top row) and between 5-min and 10-min (bottom row) for the data obtained from individual ID 1 (see Fig. B.2). We used the formula $\rho = 100 \times \frac{x_5 - x_i}{x_5}$ to compute the relative change when $i = 2, 10$ and x_t indicates the measure involved at a t-min resolution. We observe that most of the measures are relatively insensitive to the resolution and, as expected, are generally larger for the 2-min resolution (hence the mostly negative values since more data is involved) and are always smaller for the 5-min (since 10 is divisible by 5 so the 10-min data is a proper subset of the 5-min data, which implies that the relative change in values will all be non-negative). Of course, we could have carried out an analysis using the finer 2-min analyses. This extra level of analysis is recommended when the focus is on the movement ecology of the population under consideration rather than just on illustrating our methodology, as we did in our main text.

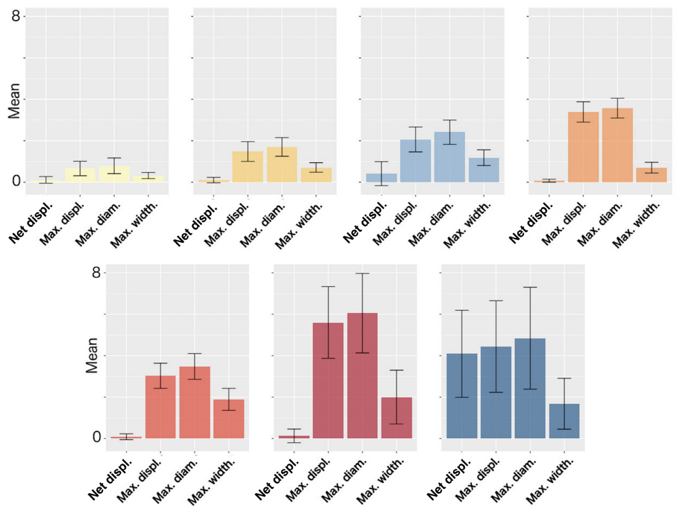

Figure B.4 Mean±sd of the four measurements for each cluster, colored accordingly to our selected color scheme (see Table 1). Top row, from the left: clusters 1,2,3 and 4. Bottom row, from the left: clusters 5, 6 and 7.

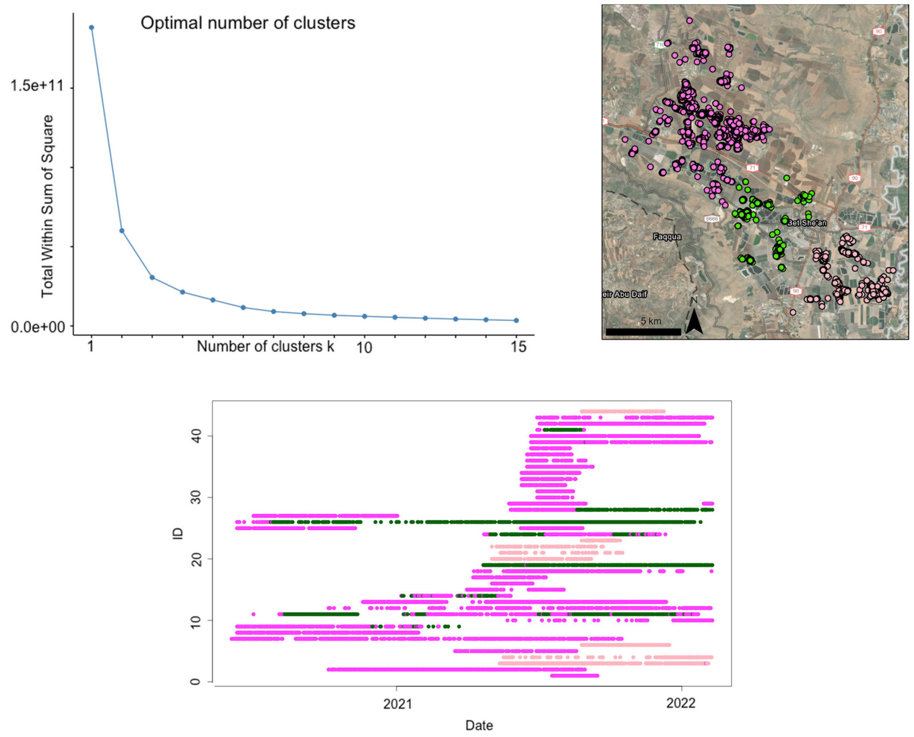

Figure B.5 Top left panel: elbow method for choosing the number of clusters for the starting points. Top right panel: resulting clusters, shown using different colors (west: magenta, centre: darkgreen, east: pink). Bottom panel: temporal distribution of the 3 clusters, ordered by individual ID number (see Table A.1).

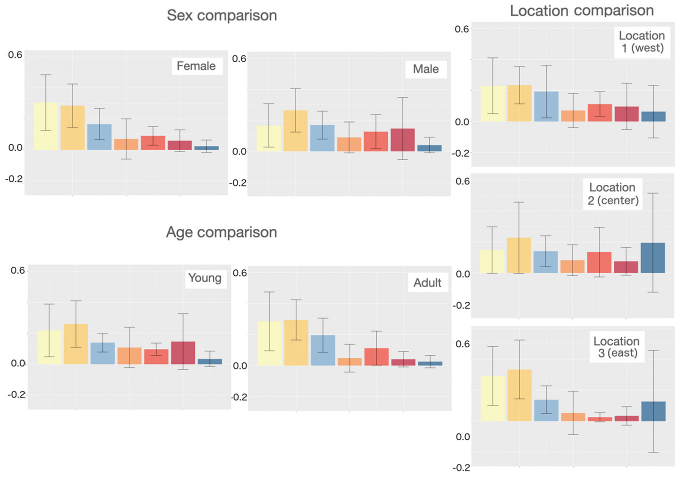

Figure B.6 The relative means±sd of the DAR frequencies of each individual, grouped by gender, age and location, and colored according to our selected scheme (see Table 1).

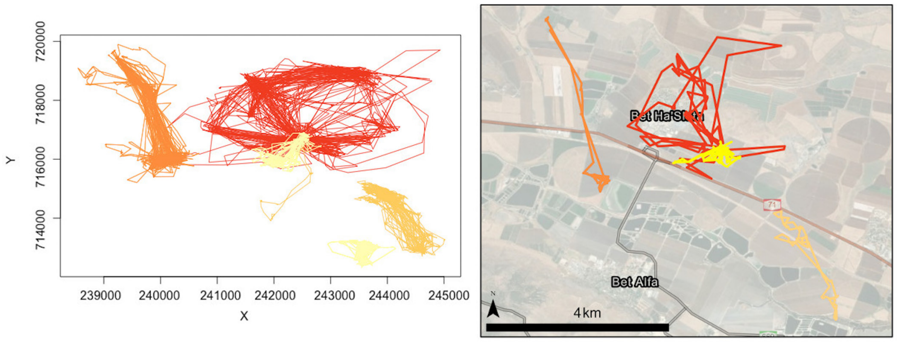

Figure B.7 Left panel: Map of most common DARs in the quadrimester June-September 2021 for each individual (colors as in Fig. 3 and Table 2). Mother (ID 11): yellow, father (ID 17): light red, fledglings: (IDs 41 and 42) dark yellow and orange respectively. (X,Y) values represent Israeli Transverse Mercator (ITM) coordinates. **Right panel**: one DAR per individual, from the most common group for each individual (individuals 11, 17, 41 and 42 on 2021-06-27, 2021-06-22, 2021-07-19, 2021-08-15 respectively).

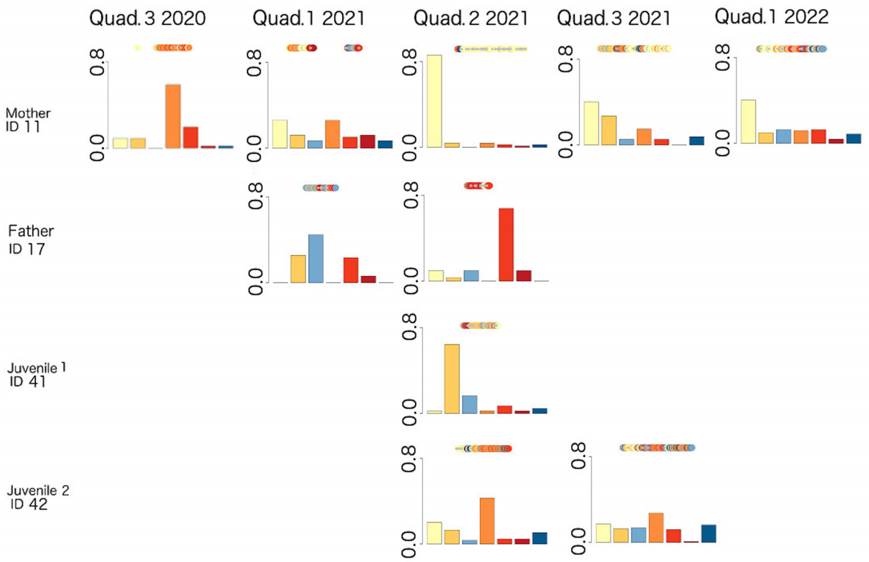

Figure B.8 Family 2: temporal DAR distribution and progression by quadrimester and by individuals.

Appendix C: Generalized Linear Mixed Model Analysis

Summary of the results of two generalized linear mixed models (GLMMs) are presented here in terms of plots and tables. Each model includes two random factors to account for repeated measures from the same individual (`RingID`) and temporal dependence (`Date`). Fixed effects for each model include the independent binary variables of `Sex` (male/female) and `Age` (young/adult) and their interaction. We fitted the model to the square root of the composite DAR-size variable `PC1` (the first principal component of our PCA) (Tables C1-C3), and to the square-root of the DAR measure `maximum displacement` (C4-C6) as the dependent variables. In both cases, the number of observations was 6074 DARs, and we validated that the distribution of the residuals (Fig. C1) was approximately normal.

Tables C1 and C4 show the variation among random effects in the two respective fits, with 44 groups for `RingID` and 615 groups for `Date`. Tables C2 and C5 show the effect size of the fixed effects. Tables C3 and C6 show Type III Analysis of Variance for the two fits respectively, using Satterthwaite's method. These results (Fig. C2) show that the two fixed effects have a significant impact on the two dependent variables, but not their interactions. Significance codes in the following tables C.2-3 & 5-6 are *** for $p < 0.001$, ** for $p < 0.01$, and * for $p < 0.05$.

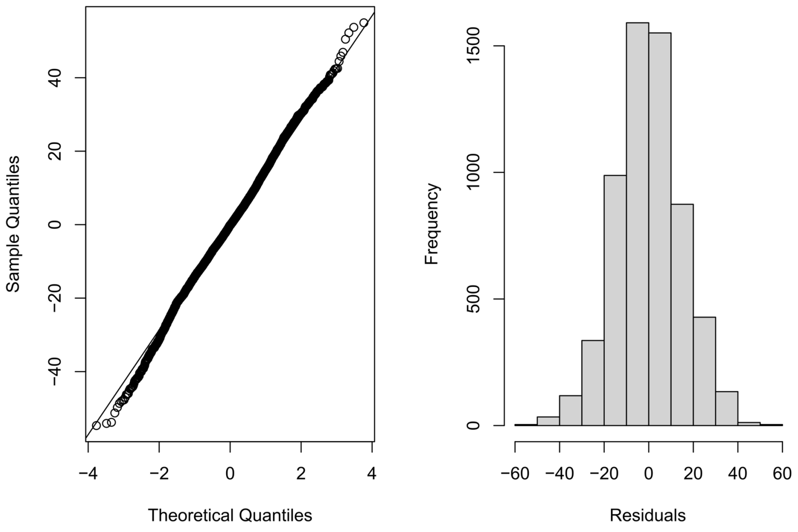

Figure C.1 Q-Q (left panel) and a histogram plot (right panel) of a residual analysis of the distribution of the `PC1` composite DAR-size data after performing a square-root transformation. Overall, the residual distribution fits a normal distribution in accordance with model assumptions.

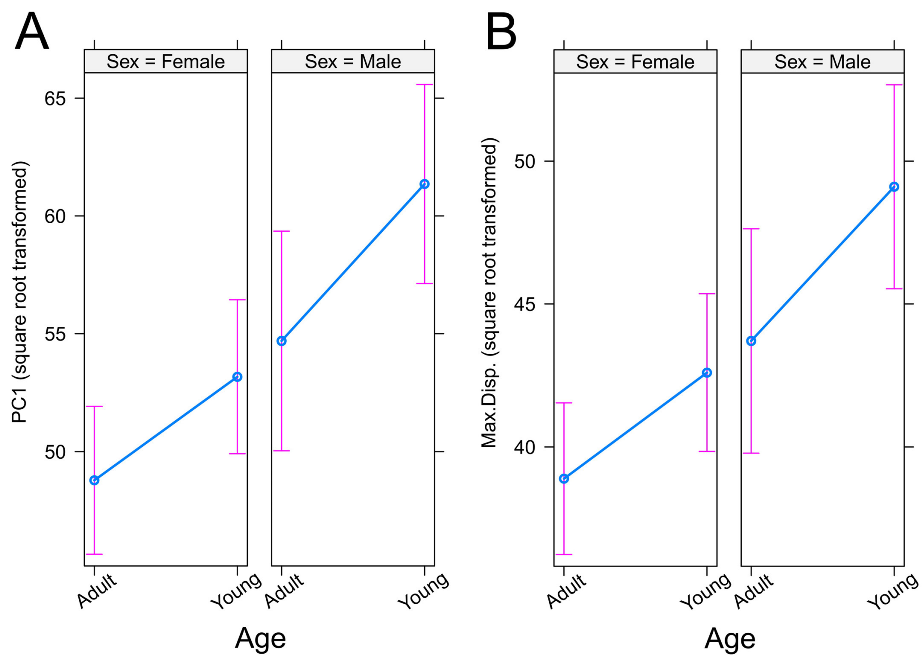

Figure C.2 Model predictions for the effects of `Age` (young/adult), `Sex`, and their interactions from generalized linear mixed models (GLMMs) of the square-root of **(A).** the composite DAR-size variable `PC1` (the first principal component of our PCA) and **(B).** the DAR measure `maximum displacement` as the dependent variables.

Table C.1 Random effects estimations from modeling the square-root of the composite DAR-size variable `PC1` (the first principal component of our PCA) with `Date` and `RingID` as random factors.

Groups	Name	Variance	Std.Dev.
**Date**	(Intercept)	28.88	5.37
**RingID**	(Intercept)	60.87	60.87
**Residual**		235.95	15.36

Table C.2 Fixed effects estimations from modeling the square-root of the composite DAR-size variable `PC1` (the first principal component of our PCA) as a function of `Age`, `Sex`, and their interaction. Reference categories were `Adult` and `Female`.

	Estimate	Std. Error	df	t value	Pr(>|t|)	
**(Intercept)**	48.78	1.60	49.88	30.47	<2e-16	***
**AgeYoung**	4.39	0.96	2979.34	4.54	5.72e-06	***
**SexMale**	5.91	2.82	61.17	2.09	0.040	*
**AgeYoung:SexMale**	2.27	2.40	572.63	0.94	0.346	

Table C.3 Type III Analysis of Variance Table with Satterthwaite's method for modeling square-root of the composite DAR-size variable `PC1` (the first principal component of our PCA) as a function of `Age`, `Sex`, and their interaction.

	Sum Sq	Mean Sq	NumDF	DenDF	F value	Pr(>F)	
**Age**	4704.7	4704.7	1	546.38	19.94	9.717e-06	***
**Sex**	1889.8	1889.8	1	43.80	8.01	0.007	**
**Age:Sex**	209.7	209.7	1	572.63	0.89	0.346	

Table C.4 Random effects estimations from modeling the square-root of the DAR measure `maximum displacement` with `Date` and `RingID` as random factors.

Groups	Name	Variance	Std.Dev.
**Date**	(Intercept)	18.02	4.24
**RingID**	(Intercept)	43.98	6.63
**Residual**		160.51	12.67

Table C.5 Fixed effects estimations from modeling the square-root of the DAR measure `maximum displacement` as a function of `Age`, `Sex`, and their interaction. Reference categories were Adult and Female.

	Estimate	Std. Error	df	t value	Pr(>|t|)	
**(Intercept)**	38.89	1.35	48.89	28.72	<2e-16	***
**AgeYoung**	3.71	0.79	3060.93	4.67	3.07e-06	***
**SexMale**	4.82	2.38	60.41	2.02	0.047	*
**AgeYoung:SexMale**	1.68	1.99	613.05	0.84	0.399	

Table C.6 Type III Analysis of Variance Table with Satterthwaite's method for modeling square-root of the DAR measure `maximum displacement` as a function of `Age`, `Sex`, and their interaction.

	Sum Sq	Mean Sq	NumDF	DenDF	F value	Pr(>F)	
**Age**	3164.9	3164.9	1	586.07	19.72	1.073e-05	***
**Sex**	1152.3	1152.3	1	43.48	7.18	0.010	*
**Age:Sex**	114.1	114.1	1	613.05	0.7109	0.399	
